# RGB-D salient object detection: A survey

**DOI:** 10.1007/s41095-020-0199-z

**Published:** 2021-01-07

**Authors:** Tao Zhou, Deng-Ping Fan, Ming-Ming Cheng, Jianbing Shen, Ling Shao

**Affiliations:** 1 Inception Institute of Artificial Intelligence (IIAI), Abu Dhabi, United Arab Emirates; 2 CS, Nankai University, Tianjin, 300350 China

**Keywords:** RGB-D, saliency, light fields, benchmarks

## Abstract

Salient object detection, which simulates human visual perception in locating the most significant object(s) in a scene, has been widely applied to various computer vision tasks. Now, the advent of depth sensors means that depth maps can easily be captured; this additional spatial information can boost the performance of salient object detection. Although various RGB-D based salient object detection models with promising performance have been proposed over the past several years, an in-depth understanding of these models and the challenges in this field remains lacking. In this paper, we provide a comprehensive survey of RGB-D based salient object detection models from various perspectives, and review related benchmark datasets in detail. Further, as light fields can also provide depth maps, we review salient object detection models and popular benchmark datasets from this domain too. Moreover, to investigate the ability of existing models to detect salient objects, we have carried out a comprehensive attribute-based evaluation of several representative RGB-D based salient object detection models. Finally, we discuss several challenges and open directions of RGB-D based salient object detection for future research. All collected models, benchmark datasets, datasets constructed for attribute-based evaluation, and related code are publicly available at https://github.com/taozh2017/RGBD-SODsurvey.

### 1 Introduction

#### 1.1 Background

Salient object detection aims to locate the most visually prominent object(s) in a given scene [1]. It plays a key role in a range of real-world applications, such as stereo matching [2], image understanding [3], co-saliency detection [4], action recognition [5], video detection and segmentation [6–9], semantic segmentation [10, 11], medical image segmentation [12–14], object tracking [15, 16], person re-identification [17, 18], camouflaged object detection [19], image retrieval [20], etc. Although significant progress has been made in the salient object detection field over the past several years [21–35], there is still room for improvement when faced with challenging factors, such as complicated backgrounds or varying lighting conditions in the scenes. One way to overcome such challenges is to employ depth maps, which provide complementary spatial information to that from RGB images and have become easier to capture due to the ready availability of depth sensors (e.g., Microsoft Kinect).

Recently, RGB-D based salient object detection has gained increasing attention, and various methods have been developed [38, 45]. Early RGB-D based salient object detection models tended to extract handcrafted features and then fused the RGB image and depth map. For example, Lang et al. [46], the first work on RGB-D based salient object detection, utilized Gaussian mixture models to model the distribution of depth-induced saliency. Ciptadi et al. [47] extracted 3D layout and shape features from depth measurements. Several methods [48–50] measure depth contrast using depth differences between different regions. In Ref. [51], a multi-contextual contrast model including local, global, and background contrast was developed to detect salient objects using depth maps. More importantly, however, this work also provided the first large-scale RGB-D dataset for salient object detection. Despite the effectiveness of traditional methods using handcrafted features, their low-level features tend

1 Inception Institute of Artificial Intelligence (IIAI), Abu Dhabi, United Arab Emirates. E-mail: dengpfan@gmail.com (✉).
2 CS, Nankai University, Tianjin 300350, China.
Manuscript received: 2020-07-31; accepted: 2020-10-07

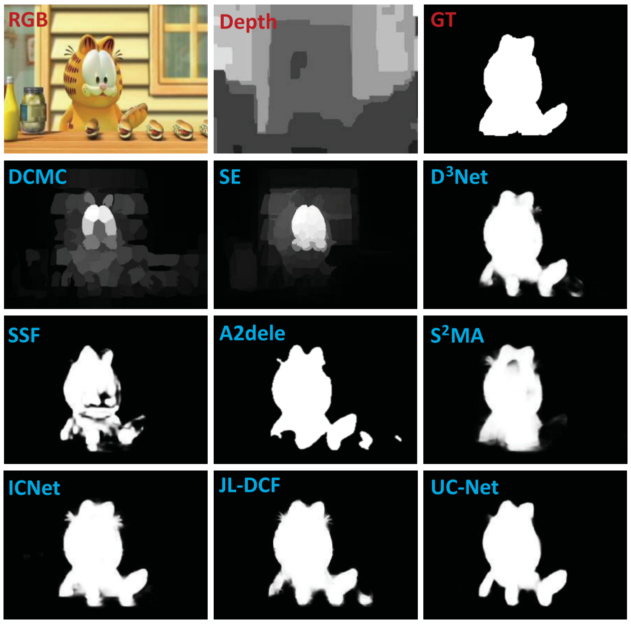

**Fig. 1** RGB-D based salient object prediction on a sample image using two classical models: DCMC [36] and SE [37], and seven state-of-the-art deep models: $D^3$Net [38], SSF [39], A2dele [40], $S^2$MA [41], ICNet [42], JL-DCF [43], and UC-Net [44].

to limit generalization ability, and they lack the high-level reasoning required for complex scenes. To address these limitations, several deep learning-based RGB-D salient object detection methods [38] have been developed, with improved performance. DF [52] was the first model to introduce deep learning technology into the RGB-D based salient object detection task. More recently, various deep learning-based models [41–44, 53–55] have focused on exploiting effective multi-modal correlations and multi-scale or level information to boost salient object detection performance. To more clearly describe the progress in the RGB-D based salient object detection field, we provide a brief chronology in Fig. 2.

In this paper, we provide a comprehensive survey of RGB-D based salient object detection, aiming to thoroughly cover various aspects of models used for this task and to provide insightful discussions of the challenges and open directions for future work. We also review a related topic, light field salient object detection, as light fields can also provide additional information (including focal stacks, all-focus images, and depth maps) to boost the performance of salient object detection. Further, we provide a comprehensive comparative evaluation of existing RGB-D based salient object detection models and discuss their main advantages.

#### 1.2 Related reviews and surveys

Several surveys consider salient object detection. For example, Borji et al. [59] provided a quantitative evaluation of 35 state-of-the-art non-deep-learning saliency detection methods. Cong et al. [60] reviewed several different saliency detection models, including RGB-D based salient object detection, co-saliency detection, and video salient object detection. Zhang et al. [61] provided an overview of co-saliency detection and reviewed its history, and summarized several benchmark algorithms in this field. Han et al. [62] reviewed recent progress in salient object detection, including models, benchmark datasets, and evaluation metrics, as well as discussing the underlying connection between general object detection, salient object detection, and category-specific object detection. Nguyen et al. [63] reviewed

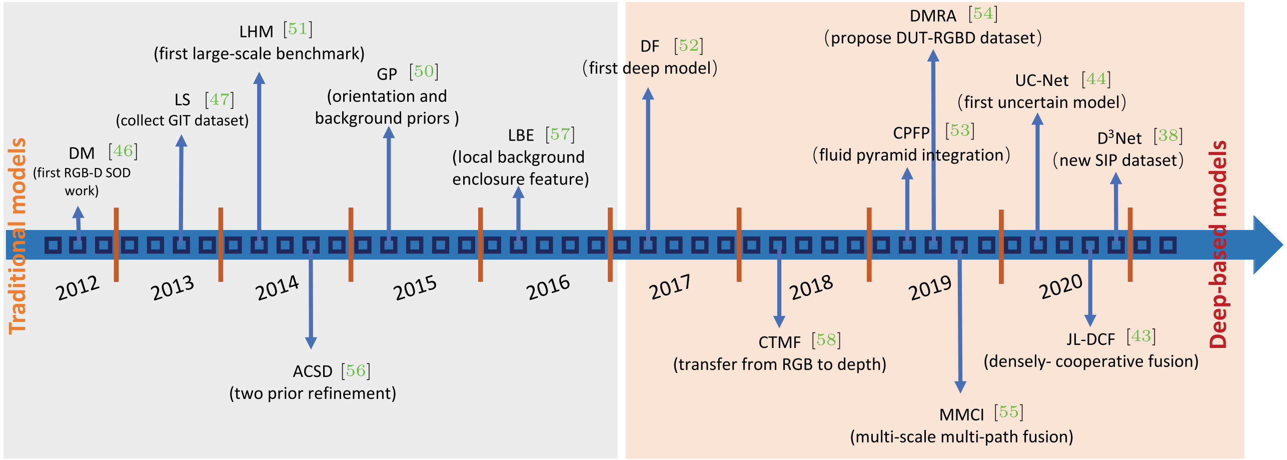

**Fig. 2** Brief chronology of RGB-D based salient object detection. First came the DM model, proposed in 2012 [46]. Deep learning techniques have been widely applied since 2017. See Section 2.

various works related to saliency applications and provide insightful discussions of the role of saliency in each. Borji et al. [64] provided a comprehensive review of recent progress in salient object detection and discussed related topics, including generic scene segmentation, saliency for fixation prediction, and object proposal generation. Fan et al. [1] provided a comprehensive evaluation of several state-of-the-art CNN-based salient object detection models, and proposed a high quality salient object detection dataset, **SOC** (see: http://dpfan.net/socbenchmark/). Zhao et al. [65] reviewed various deep learning-based object detection models and algorithms in detail, as well as various specific tasks, including salient object detection. Wang et al. [66] focused on reviewing deep learning-based salient object detection models. Unlike previous salient object detection surveys, in this paper, we focus on reviewing RGB-D based salient object detection models and benchmark datasets.

#### 1.3 Contributions and organization

Our contributions and organization are:

- the first systematic review of RGB-D based salient object detection models considering different perspectives. We classify existing RGB-D salient object detection models as traditional or deep methods, fusion-wise methods, single-stream or multi-stream methods, and attention-aware methods (Section 2);
- a review of nine RGB-D datasets commonly used in this field, giving details for each (Section 3). We also provide a comprehensive, attribute-based evaluation of several representative RGB-D based salient object detection models (Section 5);
- the first survey of light field salient object detection models and benchmark datasets (Section 4);
- a thorough investigation of challenges facing RGB-D based salient object detection, and the relationship between salient object detection and other topics, shedding light on potential directions for future research (Section 6);

Conclusions are drawn in Section 7.

### 2 RGB-D based salient object detection models

#### 2.1 Approach

Over the past few years, several RGB-D based salient object detection methods have been developed; they provide promising performance. These models are summarized in Tables 1–4. Further information can be found at http://dpfan.net/d3netbenchmark/. To review these RGB-D based salient object detection

**Table 1** Summary of RGB-D based salient object detection methods published from 2012 to 2016

| No. | Year | Method | Pub. | Training set | Backbone | Description |
|---|---|---|---|---|---|---|
| 1 | 2012 | DM [46] | ECCV | Without | Without | Models the correlation between saliency and depth by approximating the joint density using Gaussian mixture models |
| 2 | 2012 | RCM [67] | ICCSE | Without | Without | Develops a region contrast based salient object detection model with depth cues |
| 3 | 2013 | LS [47] | BMVC | Without | Without | Extends the dissimilarity framework to model the joint interaction between depth cues and RGB images |
| 4 | 2013 | RC [48] | BMVC | Without | Without | Derives RGB-D saliency by formulating a 3D saliency model based on the region contrast of the scene and fuses it using SVM |
| 5 | 2013 | SOS [68] | NEURO | Without | Without | Incorporates depth cues for salient object segmentation by suppressing background regions |
| 6 | 2014 | SRDS [69] | ICDSP | Without | Without | Integrates depth and depth weighted color contrast with spatial compactness of color distribution |
| 7 | 2014 | LHM [51] | ECCV | Without | Without | Uses a multi-stage RGB-D algorithm to combine both depth and appearance cues to segment salient objects |
| 8 | 2014 | DESM [49] | ICIMCS | Without | Without | Combines three saliency cues: color contrast, spatial bias, and depth contrast |
| 9 | 2014 | ACSD [56] | ICIP | Without | Without | Measures a point's saliency by how much it stands out from the surroundings, and has two priors (regions nearer to viewers are more salient and salient objects tend to be located at the center) |
| 10 | 2015 | GP [50] | CVPRW | Without | Without | Explores orientation and background priors for detecting salient objects, and uses PageRank and MRFs to optimize the saliency maps |
| 11 | 2015 | SFP [70] | ICIMCS | Without | Without | Develops a RGB-D based salient object detection approach using saliency fusion and propagation |
| 12 | 2015 | DIC [71] | TVC | Without | Without | Fuses the saliency maps from color and depth to generate a noise-free salient patch, and utilizes random walk algorithm to infer the object boundary |
| 13 | 2015 | SRD [72] | ICRA | Without | Without | Designs a graph-based segmentation to identify homogeneous regions using color and depth cues |
| 14 | 2015 | MGMR [73] | ICIP | Without | Without | Designs a mutual guided manifold ranking strategy to achieve salient object detection |
| 15 | 2015 | SF [74] | CAC | Without | Without | Proposes to automatically select discriminative features using decision trees for better performance |
| 16 | 2016 | PRC [75] | ACCESS | Without | Without | Saliency fusion and progressive region classification are used to optimize depth-aware saliency models |
| 17 | 2016 | LBE [57] | CVPR | Without | Without | Uses a local background enclosure to capture the spread of angular directions |

(Continued)

| No. | Year | Method | Pub. | Training set | Backbone | Description |
|---|---|---|---|---|---|---|
| 18 | 2016 | SE [37] | ICME | Without | Without | Utilizes cellular automata to propagate the initial saliency map and then generate the final saliency prediction result |
| 19 | 2016 | DCMC [36] | SPL | Without | Without | Develops a new measure to evaluate the reliability of depth maps for reducing the influence of poor-quality depth maps on saliency detection |
| 20 | 2016 | BF [76] | ICPR | Without | Without | Fuses contrasting features from RGB and depth images with a Bayesian framework |
| 21 | 2016 | DCI [77] | ICASSP | Without | Without | Adopts the original depth map to subtract the fitted surface for generating a contrast increased map |
| 22 | 2016 | DSF [78] | ICASSP | Without | Without | Develops a multi-stage depth-aware saliency model for salient object detection |
| 23 | 2016 | GM [79] | ACCV | Without | Without | Combines color and depth-based contrast features using a generative mixture model |

models in detail, we consider them from different perspectives as follows. (1) As *traditional or deep models*, according to whether manual features or deep features are used for feature extraction. It is helpful for readers to understand the historical development of RGB-D salient object detection models. (2) According to *fusion model*: it is critical to effectively fuse RGB and depth images in this task, so we review different fusion strategies to understand their effectiveness. (3) As *single- or multi-stream models*: using a single stream can reduce the number of parameters, but the final result may not be optimal; multiple streams may require more parameters. It is helpful to understand the balance between the amount of calculation and accuracy of different models. (4) According to *attention awareness*. Attention mechanisms have widely been applied to various visual tasks including salient object detection. We review related works on RGB-D salient object detection to analyze how different models use attention awareness. Alternative designs of attention modules may be useful in future work.

### 2.2 Traditional and deep models

#### *2.2.1 Traditional models*

Using depth cues, several useful attributes, such as boundaries, shape attributes, surface normals, etc.,

**Table 2** Summary of RGB-D based salient object detection methods published from 2017 to 2018

| No. | Year | Method | Pub. | Training set | Backbone | Description |
|---|---|---|---|---|---|---|
| 24 | 2017 | HOSO [80] | DICTA | Without | Without | Combines surface orientation distribution contrast with color and depth contrast |
| 25 | 2017 | $M^3$Net [81] | IROS | NLPR(0.65k), NJUD(1.4k) | VGG-16 | Designs a multi-path multi-modal fusion strategy to integrate RGB and depth images in a task-motivated and adaptive way |
| 26 | 2017 | MFLN [82] | ICCVS | NLPR(0.65k), NJUD(1.4k) | AlexNet | Leverages a CNN to learn high-level representations for depth maps, and uses a multi-modal fusion network to integrate RGB and depth representations for RGB-D based salient object detection |
| 27 | 2017 | BED [83] | ICCVW | NLPR(0.6k), NJUD(1.2k) | GoogleNet | Uses a CNN to integrate top-down and bottom-up information for RGB-D based salient object detection, and uses a mid-level feature representation to capture background enclosure |
| 28 | 2017 | CDCP [84] | ICCVW | Without | Without | Proposes a novel RGB-D salient object detection algorithm using a center dark channel prior to boost performance |
| 29 | 2017 | TPF [85] | ICCVW | Without | Without | Leverages stereopsis to generate optical flow, which can provide an additional cue (depth cue) for producing the final detection result |
| 30 | 2017 | MFF [86] | SPL | Without | Without | Uses a multistage fusion framework to integrate multiple visual priors from the RGB image and depth cue for salient object detection |
| 31 | 2017 | MDSF [87] | TIP | NLPR(0.5k), NJUD(1.5k) | Without | Proposes a RGB-D salient object detection framework via a multi-scale discriminative saliency fusion strategy, and utilizes bootstrap learning to achieve the salient object detection task |
| 32 | 2017 | DF [52] | TIP | NLPR(0.75k), NJUD(1.0k) | Without | Feeds RGB and depth features into a CNN architecture to derive the saliency confidence value, and uses Laplacian propagation to produce the final detection result |
| 33 | 2017 | MCLP [88] | TCYB | Without | Without | Utilizes the additional depth maps and employs the existing RGB saliency map as an initialization using a refinement-cycle model to obtain the final co-saliency map |
| 34 | 2018 | ISC [89] | SIVP | Without | Without | Fuses salient features using both bottom-up and top-down saliency cues |
| 35 | 2018 | HSCS [90] | TMM | Without | Without | Utilizes a hierarchical sparsity reconstruction and energy function refinement for RGB-D based co-saliency detection |
| 36 | 2018 | ICS [91] | TIP | Without | Without | Exploits the constraint correlation among multiple images and introduces depth maps into the co-saliency model |
| 37 | 2018 | CTMF [58] | TCYB | NLPR(0.65k), NJUD(1.4k) | VGG-16 | Transfers the structure of the deep color network to be applicable for the depth modality and fuses both modalities to produce the final saliency map |
| 38 | 2018 | PCF [92] | CVPR | NLPR(0.65k), NJUD(1.4k) | VGG-16 | Designs the first multi-scale fusion architecture and a novel complementarity-aware fusion module to fuse both cross-modal and cross-level features |
| 39 | 2018 | SCDL [93] | ICDSP | NLPR(0.75k), NJUD(1.0k) | VGG-16 | Designs a new loss function to increase the spatial coherence of salient objects |
| 40 | 2018 | ACCF [94] | IROS | NLPR(0.65k), NJUD(1.4k) | VGGNet | Adaptively selects complementary features from different modalities at each level, and then performs more informative cross-modal cross-level combinations |
| 41 | 2018 | CDB [95] | NEURO | Without | Without | Utilizes a contrast prior and depth-guided-background prior to construct a 3D stereoscopic saliency model |

**Table 3** Summary of RGB-D based salient object detection models published in 2019 and 2020

| No. | Year | Method | Pub. | Training set | Backbone | Description |
|---|---|---|---|---|---|---|
| 42 | 2019 | SSRC [96] | NEURO | NLPR(0.65k), NJUD(1.4k) | VGG-16 | Uses a single-stream recurrent convolutional neural network with a four-channel input and DRCNN subnetwork |
| 43 | 2019 | MLF [97] | SPL | NJUD(1.588k) | VGG-16 | Designs a salient object-aware data augmentation method to expand the training set |
| 44 | 2019 | TSRN [98] | ICIP | NJUD(1.387k) | VGG-16 | Designs a fusion refinement module to integrate output features from different modalities and resolutions |
| 45 | 2019 | DIL [99] | MTAP | NLPR(0.5k), NJUD(0.5k) | Without | Designs a consistency integration strategy to generate an image pre-segmentation result that is consistent with the depth distribution |
| 46 | 2019 | CAFM [100] | TSMC | NUS [46], NCTU [101] | VGG-16 | Utilizes a content-aware fusion module to integrate global and local information |
| 47 | 2019 | PDNet [102] | ICME | NLPR(0.5k), NJUD(1.5k) | VGG-16 | Adopts a prior-model guided master network to process RGB information, which is pre-trained on the conventional RGB dataset to overcome the limited size |
| 48 | 2019 | MMCI [55] | PR | NLPR(0.65k), NJUD(1.4k) | VGG-16 | Improves the traditional two-stream architecture by diversifying the multi-modal fusion paths and introducing cross-modal interactions in multiple layers |
| 49 | 2019 | TANet [103] | TIP | NLPR(0.65k), NJUD(1.4k) | VGG-16 | Uses a three-stream multi-modal fusion framework to explore cross-modal complementarity in both the bottom-up and top-down processes |
| 50 | 2019 | DCMF [104] | TCYB | NLPR(0.65k), NJUD(1.4k) | VGG-16 | Formulates a CNN-based cross-modal transfer learning problem for depth-induced salient object detection, and uses a dense cross-level feedback strategy to exploit cross-level interactions |
| 51 | 2019 | DGT [105] | TCYB | Without | Without | Exploits depth cues and provides a general transformation model from RGB saliency to RGB-D saliency |
| 52 | 2019 | LSF [45] | arXiv | NLPR(0.65k), NJUD(1.4k) | VGG | Designs an RGB-D system with three key components, including modality-specific representation learning, complementary information selection, and cross-modal complements fusion |
| 53 | 2019 | AFNet [106] | ACCESS | NLPR(0.65k), NJUD(1.4k) | VGG-16 | Learns a switch map that is used to adaptively fuse the predicted saliency maps from the RGB and depth modality |
| 54 | 2019 | EPM [107] | ACCESS | Without | Without | Develops an effective propagation mechanism for RGB-D co-saliency detection |
| 55 | 2019 | CPFP [53] | CVPR | NLPR(0.65k), NJUD(1.4k) | VGG-16 | Uses a contrast-enhanced network to obtain the one-channel enhanced map, and designs a fluid pyramid integration module to fuse cross-modal cross-level features in a pyramid style |
| 56 | 2019 | DMRA [54] | ICCV | NLPR(0.7k), NJUD(1.485k) | VGG-19 | Designs a depth-induced multiscale recurrent attention network for salient object detection, including a depth refinement block and a recurrent attention module |
| 57 | 2019 | DSD [108] | JVCIR | NLPR(0.5k), NJUD(1.5k) | VGG-16 | Uses a saliency fusion network to adaptively fuse both the color and depth saliency maps |
| 58 | 2020 | DPANet [109] | arXiv | NLPR(0.65k), NJUD(1.4k), DUT(0.8k) | ResNet-50 | Uses a saliency-orientated depth perception module to evaluate the potentiality of depth maps and reduce effects of contamination |
| 59 | 2020 | SSDP [110] | arXiv | NLPR(0.7k), NJUD(1.485k), DUT(0.8k) | VGG-19 | Makes use of existing labeled RGB saliency datasets together with unlabeled RGB-D data to boost salient object detection performance |
| 60 | 2020 | AttNet [111] | IVC | NLPR(0.65k), NJUD(1.4k) | VGG-16 | Deploys attention maps to boost the salient objects' location and pays more attention to the appearance information |
| 61 | 2020 | — [112] | NEURO | NLPR(0.65k), NJUD(1.4k) | VGG-16 | Uses an adaptive gated fusion module via a GAN to obtain a better fused saliency map from RGB images and depth cues |
| 62 | 2020 | CoCNN [113] | PR | STERE, NJUD | VGG-16 | Fuses color and disparity features from low to high layers in a unified deep model |
| 63 | 2020 | cmSalGAN [114] | TMM | NLPR(0.65k), NJUD(1.4k) | ResNet-50 | Aims to learn an optimal view-invariant and consistent pixel-level representation for both RGB and depth images using an adversarial learning framework |
| 64 | 2020 | PGHF [115] | ACCESS | NLPR(0.65k), NJUD(1.4k) | VGG-16 | Leverages powerful representations learned from large-scale RGB datasets to boost the model ability |

can be explored to boost the identification of salient objects in complex scenes. Over the past several years, many traditional RGB-D models based on handcrafted features have been developed [36, 37, 47–51, 56, 57, 69–71, 75, 82–84, 95]. For example, the early work in Ref. [47] focused on modeling the interaction between layout and shape features generated from the RGB image and depth map. The representative work in Ref. [51] developed a novel multi-stage RGB-D model, and constructed the first large-scale RGB-D benchmark dataset, NLPR.

#### *2.2.2 Deep models*

The above traditional methods suffer from unsatisfactory salient object detection performance due to the limited expressiveness of handcrafted features. To address this, several studies have turned to deep neural networks (DNNs) to fuse RGB-D data [39, 40, 42–44, 52–55, 83, 93, 94, 96, 102–106, 111–113, 117–119, 137]. These models can learn high-level representations to explore complex correlations between RGB images and depth cues for improving salient object detection performance. We next review some representative works.

**DF** [52] develops a novel convolutional neural network (CNN) to integrate different low-level saliency cues into hierarchical features, to effectively locate salient regions in RGB-D images. This was the first CNN-based model for RGB-D salient object detection. However, it utilizes a shallow architecture

**Table 4** Summary of RGB-D based salient object detection models published in 2020

| No. | Year | Method | Pub. | Training set | Backbone | Description |
|---|---|---|---|---|---|---|
| 65 | 2020 | BiANet [116] | TIP | NLPR(0.7k), NJUD(1.485k) | VGG-16 | Uses a bilateral attention module (BAM) to explore rich foreground and background information from depth maps |
| 66 | 2020 | ASIF-Net [117] | TCYB | NLPR(0.65k), NJUD(1.4k) | VGG-16 | Integrates the attention steered complementarity from RGB-D images and introduces a global semantic constraint using adversarial learning |
| 67 | 2020 | Triple-Net [118] | SPL | Triple-Net | ResNe-18 | Uses a triple-complementary network for RGB-D based salient object detection |
| 68 | 2020 | ICNet [42] | TIP | Triple-Net | VGG-16 | Uses a novel information conversion module to fuse high-level RGB and depth features in an interactive and adaptive way |
| 69 | 2020 | SDF [119] | TIP | NLPR, NJUD, DEC, LFSD(1.5k) | VGG-16 | Proposes a exemplar-driven method to estimate relatively trustworthy depth maps, and uses a selective deep saliency fusion network to effectively integrate RGB images, original depths, and newly estimated depths |
| 70 | 2020 | GFNet [120] | SPL | NLPR(0.8k), NJUD(1.588k) | Res2Net | Designs a gate fusion block to regularize feature fusion |
| 71 | 2020 | RGBS [121] | MTAP | NLPR(0.65k), NJUD(1.4k) | VGG-16 | Utilizes a GAN to generate the saliency map |
| 72 | 2020 | $D^3$Net [38] | TNNLS | NLPR(0.7k), NJUD(1.485k) | VGG-16 | Uses a depth purifier unit and a three-stream feature learning module to employ low-quality depth cue filtering and cross-modal feature learning, respectively |
| 73 | 2020 | JL-DCF [43] | CVPR | NLPR(0.7k), NJUD(1.5k) | VGG-16, ResNet-101 | Uses a joint learning strategy and a densely-cooperative fusion module to achieve better salient object detection performance |
| 74 | 2020 | A2dele [40] | CVPR | NLPR(0.7k), NJUD(1.485k) | VGG-16 | Employs a depth distiller to explore ways of using network prediction and attention as two bridges to transfer depth knowledge to RGB images |
| 75 | 2020 | SSF [39] | CVPR | NLPR(0.7k), NJUD(1.485k), DUT(0.8k) | AGG-16 | Designs a complimentary interaction module to select useful representations from the RGB and depth images and then integrate cross-modal features |
| 76 | 2020 | $S^2$MA [41] | CVPR | NLPR(0.65k), NJUD(1.4k) | VGG-16 | Fuses multi-modal information via self-attention and each otherâĂŹs attention strategies, and reweights the mutual attention term to filter out unreliable information |
| 77 | 2020 | UC-Net [44] | CVPR | NLPR(0.7k), NJUD(1.5k) | VGG-16 | Uses a probabilistic RGB-D saliency detection network via a conditional VAE to generate multiple saliency maps |
| 78 | 2020 | CMWNet [122] | ECCV | NLPR(0.65k), NJUD(1.4k) | VGG-16 | Exploits feature interactions using three cross-modal cross-scale weighting modules to improve salient object detection performance |
| 79 | 2020 | HDFNet [123] | ECCV | NLPR(0.7k), NJUD(1.485k), DUT(0.8k) | VGG-16 | Designs a hierarchical dynamic filtering network to effectively make use of cross-modal fusion information |
| 80 | 2020 | CAS-GNN [124] | ECCV | NLPR(0.65k), NJUD(1.4k) | VGG-16 | Designs cascaded graph neural networks to exploit useful knowledge from RGB and depth images for building powerful feature embeddings |
| 81 | 2020 | CMMS [125] | ECCV | NLPR(0.7k), NJUD(1.485k) | VGG-16 | Proposes a cross-modality feature modulation module to enhance feature representations and an adaptive feature selection module to gradually select saliency-related features |
| 82 | 2020 | DANet [126] | ECCV | NLPR(0.65k), NJUD(1.4k) | VGG-16, VGG-19 | Develops a single-stream network combined with a depth-enhanced dual attention to achieve real-time salient object detection |
| 83 | 2020 | CoNet [127] | ECCV | NLPR(0.7k), NJUD(1.485k), DUT(0.8k) | ResNet | Develops a collaborative learning framework for RGB-D based salient object detection. Three collaborators (edge detection, coarse salient object detection and depth estimation) are utilized to jointly boost the performance |
| 84 | 2020 | BBS-Net [128] | ECCV | NLPR(0.65k), NJUD(1.4k) | VGG-16, VGG-19, ResNet-50 | Uses a bifurcated backbone strategy to learn teacher and student features, and utilizes a depth-enhanced module to excavate informative parts of depth cues |
| 85 | 2020 | ATSA [129] | ECCV | NLPR(0.7k), NJUD(1.485k), DUT(0.8k) | VGG-19 | Proposes an asymmetric two-stream architecture taking account of the inherent differences between RGB and depth data for salient object detection |
| 86 | 2020 | PGAR [130] | ECCV | NLPR(0.7k), NJUD(1.485k) | VGG-16 | Proposes a progressively guided alternate refinement network to produce a coarse initial prediction using a multi-scale residual block |
| 87 | 2020 | MCINet [131] | arXiv | NLPR(0.65k), NJUD(1.4k) | ResNet-50 | Develops a novel multi-level cross-modal interaction network for RGB-D salient object detection |
| 88 | 2020 | DRLF [132] | TIP | NLPR(0.65k), NJUD(1.4k) | VGG-16 | Develops a channel-wise fusion network to conduct multi-net and multi-level selective fusion for RGB-D salient object detection |
| 89 | 2020 | DQAM [133] | arXiv | NLPR(0.65k), NJUD(1.4k) | Without | Proposes a depth quality assessment solution to conduct "quality-aware" salient object detection for RGB-D images |
| 90 | 2020 | DQSD [134] | TIP | NLPR(0.65k), NJUD(1.4k) | VGG-19 | Integrates a depth quality aware subnet into a bi-stream structure to assess the depth quality before conducting RGB-D fusion |
| 91 | 2020 | DASNet [135] | ACM MM | NLPR(0.7k), NJUD(1.5k) | ResNet-50 | Proposes a new perspective of containing the depth constraints in the learning process rather than using depths as inputs |
| 92 | 2020 | DCMF [136] | TIP | NLPR(0.65k), NJUD(1.4k) | VGG-16, ResNet-50 | Designs a disentangled cross-modal fusion network to expose structural and content representations from RGB and depth images |

to learn the saliency map.

**PCF** [92] presents a complementarity-aware fusion module to integrate cross-modal and cross-level feature representations. It can effectively exploit complementary information by explicitly using cross-modal and -level connections and modal- and level-wise supervision to decrease fusion ambiguity.

**CTMF** [58] employs a computational model to identify salient objects from RGB-D scenes, utilizing CNNs to learn high-level representations for RGB images and depth cues, while simultaneously exploiting the complementary relationships and joint representation. This model transfers the structure of the model from the source domain (RGB images) to the target domain (depth maps).

**CPFP** [53] proposes a contrast-enhanced network to produce an enhanced map, and presents a fluid pyramidal integration module to effectively fuse cross-modal information in a hierarchical manner. As depth cues tend to suffer from noise, a feature-enhanced module is used to learn enhanced depth cues for to effectively boost salient object detection performance.

**UC-Net** [44] proposes a probabilistic RGB-D based salient object detection network via conditional variational autoencoders to model human annotation uncertainty. It generates multiple saliency maps for each input image by sampling the learned latent space. This was the first work to investigate uncertainty in RGB-D based salient object detection, and was inspired by the data labeling process. It leverages diverse saliency maps to improve the final salient object detection performance.

#### 2.3 Fusion approach

For RGB-D based salient object detection models, it is important to effectively fuse RGB images and depth maps. Existing fusion strategies can be classified as using early fusion, multi-scale fusion, or late fusion, as we now explain; also see Fig. 3.

##### *2.3.1 Early fusion*

Early fusion-based methods work in one of two ways: (i) RGB images and depth maps are directly integrated to form a four-channel input [50, 51, 87, 96], which we call *input fusion*, or (ii) RGB and depth images are first fed into separate networks and their low-level representations are combined to give a joint representation which is then fed into a subsequent network for further saliency map prediction [52]. We call this *early feature fusion*.

##### *2.3.2 Late fusion*

Late fusion-based methods can also be further divided into two families: (i) two parallel network streams are adopted to learn high-level features for RGB and depth data, respectively, which are concatenated and then used to generate the final saliency prediction [48, 58, 106]. We call this *later feature fusion*. (ii) Two parallel network streams are used to obtain independent saliency maps for RGB images and depth cues, and then the two saliency maps are concatenated to obtain a final prediction map [108]. This is called *late result fusion*.

##### *2.3.3 Multi-scale fusion*

To effectively explore the correlations between RGB images and depth maps, several methods propose a multi-scale fusion strategy [42, 43, 55, 109, 116, 122, 123, 128]. These models can be divided into two categories. The first learns the cross-modal interactions and then fuses them into a feature learning network. For example, Chen et al. [55] developed a multi-scale, multi-path fusion network to integrate RGB images and depth maps, with a cross-modal interaction (MMCI) module. This method introduces cross-modal interactions into multiple layers, which can provide additional gradients for enhancing learning of the depth stream, as well as enabling complementarity between low-level and high-level representations to be explored. The second category fuses features from RGB images and depth maps in different layers and then integrates them into a decoder network (e.g., via skip connections) to produce the final saliency detection map. Some representative works are now briefly discussed.

**ICNet** [42] proposes an information conversion module to interactively convert high-level features.

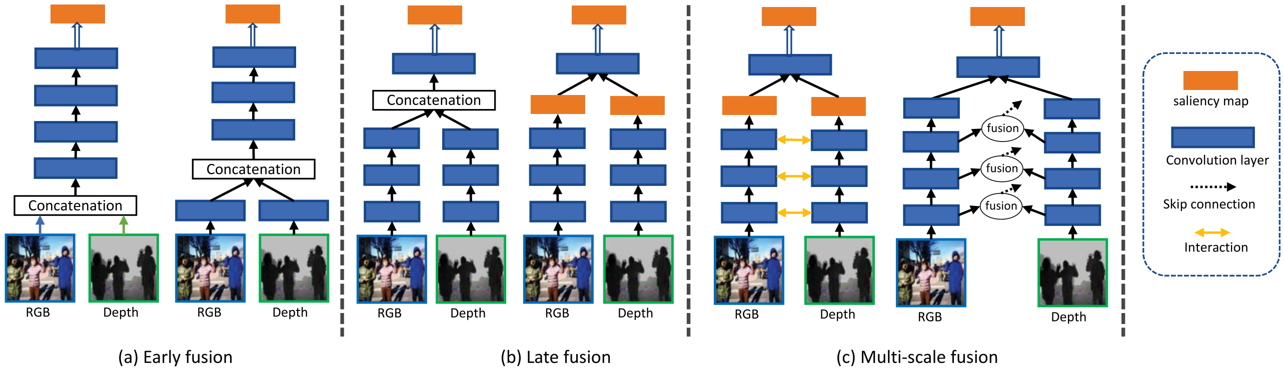

**Fig. 3** Three fusion strategies for exploring the correlation between RGB images and depth maps for RGB-D based salient object detection: (a) early fusion, (b) late fusion, and (c) multi-scale fusion.

In this model, a cross-modal depth-weighted combination (CDC) block is introduced to enhance RGB features with depth features at different levels.

**DPANet** [109] uses a gated multi-modality attention (GMA) module to exploit long-range dependencies. The GMA module can extract the most discriminatory features by utilizing a spatial attention mechanism. This model also controls the fusion rate of the cross-modal information using a gate function, which can reduce some effects caused by unreliable depth cues.

**BiANet** [116] employs a multi-scale bilateral attention module (MBAM) to capture better global information from multiple layers.

**JL-DCF** [43] treats a depth image as a special case of a color image and employs a shared CNN for both RGB and depth feature extraction. It also proposes a densely-cooperative fusion strategy to effectively combine the features learned from different modalities.

**BBS-Net** [128] uses a bifurcated backbone strategy (BBS) to split the multi-level feature representations into teacher and student features, and develops a depth-enhanced module (DEM) to explore informative parts in depth maps from the spatial and channel views.

### 2.4 Single- and multi-stream models

#### *2.4.1 Single-stream models*

Several RGB-D based salient object detection works [52, 53, 83, 87, 93, 96, 102] focus on a single-stream architecture to achieve saliency prediction. These models often fuse RGB images and depth information in the input channel or feature learning part. For example, MDSF [87] employs a multi-scale discriminative saliency fusion framework as the salient object detection model, in which four types of features from three levels are computed and then fused to obtain the final saliency map. BED [83] utilizes a CNN architecture to integrate bottom–up and top–down information for salient object detection. It incorporates multiple features, including background enclosure distribution (BED) and low level depth maps (e.g., depth histogram distance and depth contrast) to boost salient object detection performance. PDNet [102] extracts depth-based features using a subsidiary network, which makes full use of depth information to assist the main-stream network.

#### *2.4.2 Multi-stream models*

Two-stream models [54, 106, 111] have two independent branches to process RGB images and depth cues, respectively, and often generate different high-level features or saliency maps, and then incorporate them in the middle stage or at the end of the two streams. Most recent deep learning-based models [40, 42, 45, 55, 92, 104, 109, 112, 114, 117] utilize this two-stream architecture with several models capturing the correlations between RGB images and depth cues across multiple layers. Moreover, some models utilize a multi-stream structure [38, 103] and then design different fusion modules to effectively fuse RGB and depth information in order to exploit their correlations.

### 2.5 Attention models

Existing RGB-D based salient object detection methods often treat all regions equally using the extracted features in the same way, while ignoring the fact that different regions can make different contributions to the final prediction map. These methods are easily affected by cluttered backgrounds. Furthermore, some methods either regard the RGB images and depth maps as having the same status or overly rely on depth information. This prevents them from considering the importance of different domains (RGB images or depth cues). To overcome such issues, several methods introduce attention mechanisms to weight the importance of different regions or domains.

**ASIF-Net** [117] captures complementary information from RGB images and depth cues using interwoven fusion, and weights saliency regions through a deeply supervised attention mechanism.

**AttNet** [111] introduces attention maps for differentiating between salient objects and background regions to reduce the negative influence of certain low-quality depth cues.

**TANet** [103] formulates a multi-modal fusion framework using RGB images and depth maps from bottom–up and top–down views. It then introduces a channel-wise attention module to effectively fuse the complementary information from different modalities and levels.

### 2.6 Open-source implementations

Available open-source implementations of RGB-D based salient object detection models reviewed in this survey are provided in Table 5. Further source code will

**Table 5** RGB-D based salient object detection models with open-source implementations

| Year | Model | Implementation | Code link |
|---|---|---|---|
| 2014 | LHM [51] | Matlab | https://sites.google.com/site/rgbdsaliency/code |
| | DESM [49] | Matlab | https://github.com/HzFu/DES_code |
| 2015 | GP [50] | Matlab | https://github.com/JianqiangRen/Global_Priors_RGBD_Saliency_Detection |
| 2016 | DCMC [36] | Matlab | https://github.com/rmcong/Code-for-DCMC-method |
| | LBE [57] | Matlab & C++ | http://users.cecs.anu.edu.au/ u4673113/lbe.html |
| 2017 | BED [83] | Caffe | https://github.com/sshige/rgbd-saliency |
| | CDCP [84] | Matlab | https://github.com/ChunbiaoZhu/ACVR2017 |
| | MDSF [87] | Matlab | https://github.com/ivpshuhttps://github.com/ivpshu |
| | DF [52] | Matlab | https://pan.baidu.com/s/1Y-PqAjuH9xREBjfl7H45HA |
| 2018 | CTMF [58] | Caffe | https://github.com/haochen593/CTMF |
| | PCF [92] | Caffe | https://github.com/haochen593/PCA-Fuse_RGBD_CVPR18 |
| | PDNet [102] | TensorFlow | https://github.com/cai199626/PDNet |
| 2019 | AFNet [106] | TensorFlow | https://github.com/Lucia-Ningning/Adaptive_Fusion_RGBD_Saliency_Detection |
| | CPFP [53] | Caffe | https://github.com/JXingZhao/ContrastPrior |
| | DMRA [54] | PyTorch | https://github.com/jiwei0921/DMRA |
| | DGT [105] | Matlab | https://github.com/rmcong/Code-for-DTM-Method |
| 2020 | ICNet [42] | Caffe | https://github.com/MathLee/ICNet-for-RGBD-SOD |
| | JL-DCF [43] | Pytorch, Caffe | https://github.com/kerenfu/JLDCF |
| | A2dele [40] | PyTorch | https://github.com/OIPLab-DUT/CVPR2020-A2dele |
| | SSF [39] | PyTorch | https://github.com/OIPLab-DUT/CVPR_SSF-RGBD |
| | ASIF-Net [117] | TensorFlow | https://github.com/Li-Chongyi/ASIF-Net |
| | $S^2$MA [41] | PyTorch | https://github.com/nnizhang/S2MA |
| | UC-Net [44] | PyTorch | https://github.com/JingZhang617/UCNet |
| | $D^3$Net [38] | PyTorch | https://github.com/DengPingFan/D3NetBenchmark |
| | CMWNet [122] | Caffe | https://github.com/MathLee/CMWNet |
| | HDFNet [123] | PyTorch | https://github.com/lartpang/HDFNet |
| | CMMS [125] | TensorFlow | https://github.com/Li-Chongyi/cmMS-ECCV20 |
| | CAS-GNN [124] | PyTorch | https://github.com/LA30/Cas-Gnn |
| | DANet [126] | PyTorch | https://github.com/Xiaoqi-Zhao-DLUT/DANet-RGBD-Saliency |
| | CoNet [127] | PyTorch | https://github.com/jiwei0921/CoNet |
| | DASNet [135] | PyTorch | http://cvteam.net/projects/2020/DASNet/ |
| | BBS-Net [128] | PyTorch | https://github.com/DengPingFan/BBS-Net |
| | ATSA [129] | PyTorch | https://github.com/sxfduter/ATSA |
| | PGAR [130] | PyTorch | https://github.com/ShuhanChen/PGAR_ECCV20 |
| | FRDT [138] | PyTorch | https://github.com/jack-admiral/ACM-MM-FRDT |

be kept updated at: https://github.com/taozh2017/RGBD-SODsurvey.

### 3 RGB-D datasets

With the rapid development of RGB-D based salient object detection, various datasets have been constructed over the past several years. Table 6 summarizes nine popular RGB-D datasets, and Fig. 4 shows examples of images (including RGB images, depth maps, and annotations) from these datasets. We provide details for each dataset next.

**STERE** [139]. The authors collected 1250 stereoscopic images from Flickr (http://www.flickr.com/), NVIDIA 3D Vision Live (http://photos.3dvisionlive.com/), and the Stereoscopic Image Gallery (http://www.stereophotography.com/). The most salient objects in each image were annotated by three users. All annotated images were then sorted based on the overlapping salient regions and the top 1000 images were selected to construct the final dataset. This was the first collection of stereoscopic images in this field.

**GIT** [47] consists of 80 color and depth images, collected using a mobile-manipulator robot in a real-world home environment. Each image is annotated based on pixel-level segmentation of its objects.

**DES** [49] consists of 135 indoor RGB-D images,

**Table 6** Nine RGB-D benchmark datasets, by year, place of publication (Pub.), dataset size, number of objects in the images (#Obj.), type of scene, depth sensor, and resolution. They can be downloaded from our website: http://dpfan.net/d3netbenchmark/

| No. | Dataset | Year | Pub. | Size | #Obj. | Types | Sensor | Resolution |
|---|---|---|---|---|---|---|---|---|
| 1 | **STERE** [139] | 2012 | CVPR | 1000 | ∼One | Internet | Stereo camera+sift flow | $[251-1200]\times[222-900]$ |
| 2 | **GIT** [47] | 2013 | BMVC | 80 | Multiple | Home environment | Microsoft Kinect | $640\times480$ |
| 3 | **DES** [49] | 2014 | ICIMCS | 135 | One | Indoor | Microsoft Kinect | $640\times480$ |
| 4 | **NLPR** [51] | 2014 | ECCV | 1000 | Multiple | Indoor/outdoor | Microsoft Kinect | $640\times480$, $480\times640$ |
| 5 | **LFSD** [140] | 2014 | CVPR | 100 | One | Indoor/outdoor | Lytro Illum camera | $360\times360$ |
| 6 | **NJUD** [56] | 2014 | ICIP | 1985 | ∼One | Movie/Internet/photo | FujiW3 camera+optical flow | $[231-1213]\times[274-828]$ |
| 7 | **SSD** [85] | 2017 | ICCVW | 80 | Multiple | Movies | Sun's optical flow | $960\times1080$ |
| 8 | **DUT-RGBD** [137] | 2019 | ICCV | 1200 | Multiple | Indoor/outdoor | — | $400\times600$ |
| 9 | **SIP** [38] | 2020 | TNNLS | 929 | Multiple | Person in the wild | Huawei Mate10 | $992\times744$ |

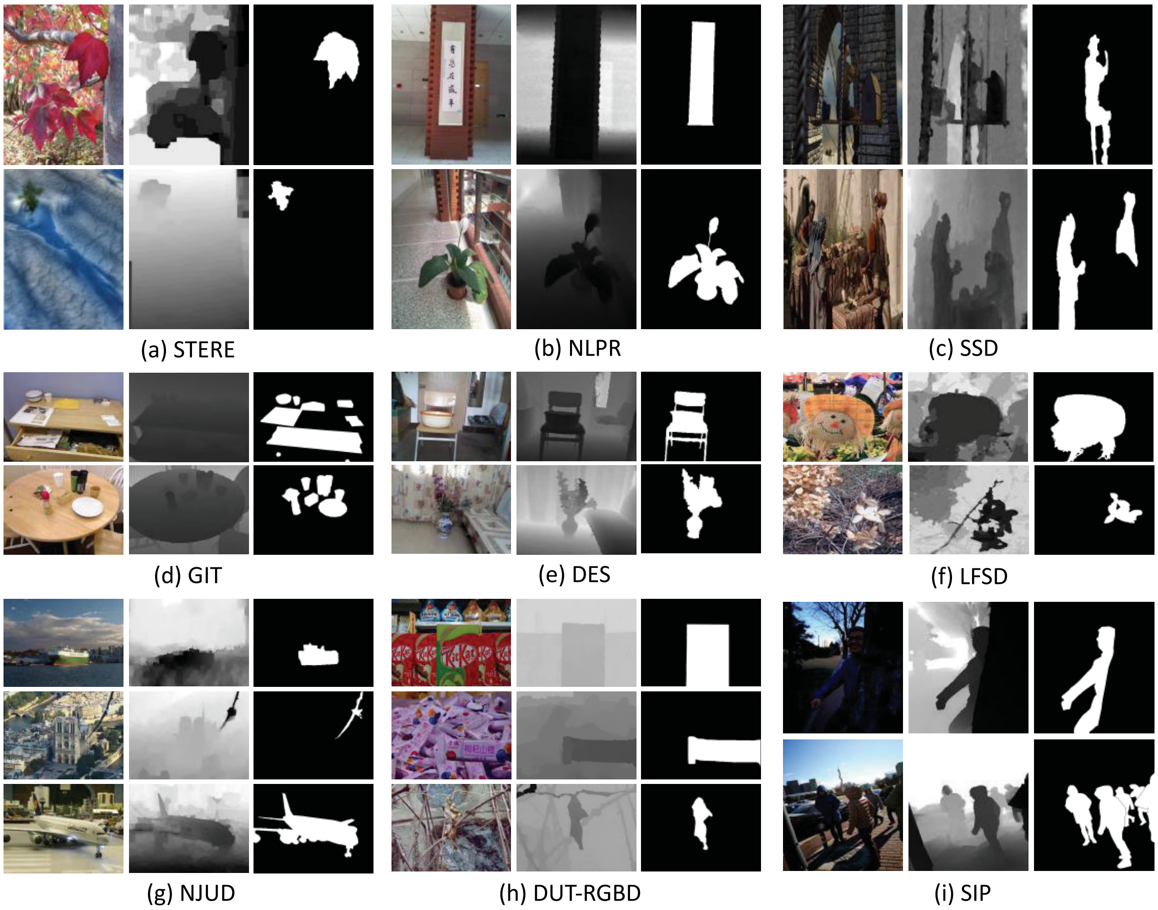

**Fig. 4** Left to right: examples of RGB images, depth maps, and annotations from nine RGB-D datasets: (a) STERE [139], (b) NLPR [51], (c) SSD [85], (d) GIT [47], (e) DES [49] , (f) LFSD [140], (g) NJUD [56], (h) DUT-RGBD [137], and (i) SIP [38].

taken by Kinect at a resolution of $640\times640$. When collecting this dataset, three users were asked to label the salient object in each image, and overlapping labeled areas were regarded as the ground truth.

**NLPR** [51] consists of 1000 RGB images and corresponding depth maps, obtained by a standard Microsoft Kinect. This dataset includes a series of outdoor and indoor locations, e.g., offices, supermarkets, campuses, streets, and so on.

**LFSD** [140] includes 100 light fields collected using a Lytro light field camera, and consists of 60 indoor and 40 outdoor scenes. To label this dataset, three individuals were asked to manually segment salient regions; the segmented results were deemed ground truth when the overlap of the three results was over 90%.

**NJUD** [56] consists of 1985 stereo image pairs, collected from the Internet, 3D movies, and photographs taken by a Fuji W3 stereo camera.

**SSD** [85] was constructed using three stereo movies and includes indoor and outdoor scenes. It includes 80 samples; each image has resolution of $960\times1080$.

**DUT-RGBD** [137] consists of 800 indoor and 400 outdoor scenes with corresponding depth images. This dataset provides several challenging factors: multiple and transparent objects, complex backgrounds, similar foregrounds to backgrounds, and low-intensity environments.

**SIP** [38] consists of 929 annotated high-resolution images, with multiple salient persons in each image. In this dataset, depth maps were captured using a smart phone (Huawei Mate10). This dataset covers diverse scenes and various challenging factors, and is annotated with pixel-level ground truth.

A detailed dataset statistical analysis (including center bias, size of objects, background objects, object boundary conditions, and number of salient objects) can be found in Ref. [38].

### 4 Saliency detection on light fields

#### 4.1 Models

##### *4.1.1 Background*

Salient object detection methods can be grouped into three categories according to the input data type: RGB, RGB-D, or light field [141]. We have already reviewed RGB-D based salient object detection models, in which depth maps provide geometric information to improve salient object detection performance to some extent. However, inaccurate or low-quality depth maps often decrease performance. To overcome this issue, light field salient object detection methods have been proposed to make use of the rich information captured by a light field. Specifically, light field data can provide an all-focus image, a focal stack, and a rough depth map [137]. A summary of light field salient object detection works is provided in Table 7; we now review them in more detail.

##### *4.1.2 Traditional and deep models*

Classic models for light field salient object detection often use superpixel-level handcrafted features [137, 140, 142–147, 149, 155]. Early work [140, 147] showed that the unique refocusing capability of light fields can provide useful focus, depth, and object identity cues, leading to several salient object detection models using light field data. For example, Zhang et al. [143] utilized a set of focal slices to compute

**Table 7** Popular light field salient object detection methods

| No. | Year | Method | Pub. | Dataset | Description |
|---|---|---|---|---|---|
| 1 | 2014 | LFS [140] | CVPR | LFSD | The first light-field saliency detection algorithm employs object identity and focus cues based on the refocusing capability of the light field |
| 2 | 2015 | WSC [142] | CVPR | LFSD | Uses a weighted sparse coding framework to learn a saliency/non-saliency dictionary |
| 3 | 2015 | DILF [143] | IJCAI | LFSD | Incorporates depth contrast to complement the disadvantage of color and uses focus-based background priors to boost the saliency detection performance |
| 4 | 2016 | RL [144] | ICASSP | LFSD | Utilizes the inherent structure information in light field images to improve saliency detection |
| 5 | 2017 | MA [145] | TOMM | HFUT, LFSD | Integrates multiple saliency cues extracted from light field images using a random-search-based weighting strategy |
| 6 | 2017 | BIF [146] | NPL | LFSD | Integrates color-based contrast, depth-induced contrast, focus map of foreground slice, and background weighted depth contrast using a two-stage Bayesian integration framework |
| 7 | 2017 | LFS [147] | TPAMI | LFSD | An extension of Ref. [140] |
| 8 | 2017 | RLM [148] | ICIVC | LFSD | Utilizes the light field relative location measurement for salient object detection on light field images |
| 9 | 2018 | SGDC [149] | CVPR | LFSD | Designs a saliency-guided depth optimization framework for multi-layer light field displays |
| 10 | 2018 | DCA [150] | FiO | LFSD | Proposes a graph model depth-induced cellular automata to optimize saliency maps using light field data |
| 11 | 2019 | DLLF [151] | ICCV | DUTLF-FS, LFSD | Utilizes a recurrent attention network to fuse each slice from the focal stack to learn the most informative features |
| 12 | 2019 | DLSD [152] | IJCAI | DUTLF-MV | Formulates saliency detection into two subproblems, including 1) light field synthesis from a single view and 2) light-field-driven saliency detection |
| 13 | 2019 | Molf [153] | NIPS | UTLF-FS | Uses a memory-oriented decoder for light field salient object detection |
| 14 | 2020 | ERNet [154] | AAAI | DUTLF-FS, HFUT, LFSD | Uses an asymmetrical two-stream architecture to overcome computation-intensive and memory-intensive challenges in a high-dimensional light field data |
| 15 | 2020 | DCA [137] | TIP | LFSD | Presents a saliency detection framework on light fields based on the depth-induced cellular automata (DCA) model. It can enforce spatial consistency to optimize the inaccurate saliency map using the DCA model |
| 16 | 2020 | RDFD [155] | MTAP | LFSD | Defines a region-based depth feature descriptor extracted from the light field focal stack to facilitate low- and high-level cues for saliency detection |
| 17 | 2020 | LFNet [141] | TIP | DUTLF-FS, LFSD, HFUT | Utilizes a light field refinement module and a light field integration module to effectively integrate multiple cues (focus, depth, and object identity) from light field images |
| 18 | 2020 | LFDCN [156] | TIP | Lytro Illum, LFSD, HFUT | Uses a deep convolutional network based on the modified DeepLab-v2 model to explore spatial and multi-view properties of light field images for saliency detection |

a background prior, and then combined it with a location prior for salient object detection. Wang et al. [146] proposed a two-stage Bayesian fusion model to integrate multiple contrasts for boosting salient object detection performance. Recently, several deep learning-based light field salient object detection models [141, 151–154, 156] have also been developed, obtaining remarkable performance. In Ref. [151], an attentive recurrent CNN was developed to fuse all focal slices, while data diversity was increased using adversarial examples to enhance model robustness. Zhang et al. [153] developed a memory-oriented decoder for light field salient object detection, which fuses multi-level features in a top–down manner using high-level information to guide low-level feature selection. LFNet [141] employs a new integration module to fuse features from light field data according to their contributions, and captures the spatial structure of a scene to improve salient object detection performance.

#### 4.2 Refinement-based models

Several refinement strategies have been used to enforce neighborhood constraints or to reduce the homogeneity of multiple modalities for salient object detection. For example, in Ref. [142], the saliency dictionary was refined using an estimated saliency map. The MA method [145] employs a two-stage saliency refinement strategy to produce the final prediction map, so that adjacent superpixels obtain similar saliency values. LFNet [141] presents an effective refinement module to reduce the homogeneity between different modalities as well to refine their dissimilarities.

#### 4.3 Light field data

Five representative datasets are widely used in existing light field salient object detection methods, as we now describe.

**LFSD** [140] consists of 100 light fields of different scenes with $360\times360$ spatial resolution, captured using a Lytro light field camera. This dataset contains 60 indoor and 40 outdoor scenes, and most scenes include only one salient object. Three individuals were asked to manually segment salient regions in each image, and ground truth was determined to occur when all three segmentation results had an overlap of over 90%. (https://sites.duke.edu/nianyi/publication/saliency-detection-on-light-field/)

**HFUT** [145] consists of 255 light fields captured using a Lytro camera. Most scenes contain multiple objects at different locations and scales, with complex background clutter. (https://github.com/pencilzhang/HFUT-Lytro-dataset)

**DUTLF-FS** [151] consists of 1465 samples, 1000 for use as a training set, and 465 for a test set. The resolution of each image is $600 \times 400$. This dataset contains several challenges, including low contrast between salient objects and cluttered backgrounds, multiple disconnected salient objects, and dark and bright lighting conditions. (https://github.com/OIPLab-DUT/ICCV2019_Deeplightfield_Saliency)

**DUTLF-MV** [152] consists of 1580 samples, 1100 for training and the remainder for testing. Images were captured by a Lytro Illum camera, and each light field consists of multi-view images and corresponding ground truth. (https://github.com/OIPLab-DUT/IJCAI2019-Deep-Light-Field-Driven-Saliency-Detection-from-A-Single-View)

**Lytro Illum** [156] consists of 640 light fields and the corresponding per-pixel ground-truth saliency maps. It includes several challenging factors, e.g., inconsistent illumination conditions, and small salient objects existing in a similar or cluttered background. (https://github.com/pencilzhang/MAC-light-field-saliency-net)

### 5 Model evaluation and analysis

#### 5.1 Evaluation metrics

We briefly review several popular metrics for salient object detection evaluation: precision-recall (PR), F-measure [59, 157], mean absolute error (MAE) [158], structural measure (S-measure) [159], and enhanced-alignment measure (E-measure) [160].

**PR**. Given a saliency map $S$, we can convert it to a binary mask $M$, and then compute the *precision* $P$ and *recall* $R$ by comparing $M$ with a ground-truth map $G$:

$$P = \frac{|M \cap G|}{|M|}, \quad R = \frac{|M \cap G|}{|G|} \tag{1}$$

A popular strategy is to partition the saliency map $S$ using a set of thresholds (from 0 to 255). For each threshold, we calculate a pair of recall and precision scores, and then combine them to obtain a PR curve that describes the performance of the model as threshold varies.

**F-measure** ($F_\beta$). The F-measure takes into account both precision and recall in a single measure, using the weighted harmonic mean:

$$F_\beta = \left(1+\beta^2\right)\frac{PR}{\beta^2 P + R} \tag{2}$$

where $\beta^2$ is set to 0.3 to emphasize precision [157]. We may again vary threshold and compute the F-measure, yielding a set of F-measure values, from which we report the maximal or average $F_\beta$.

**MAE**. This measures the average pixel-wise absolute error between a saliency map $S$ and a ground truth map $G$ for all pixels. It can be defined by

$$\mathrm{MAE} = \frac{1}{WH}\sum_{i=1}^{W}\sum_{i=1}^{H}\left|S_{i,j}-G_{i,j}\right| \tag{3}$$

where $W$ and $H$ denote the width and height of the map, respectively. MAE values are normalized to [0, 1].

**S-measure** ($S_\alpha$). To capture the importance of the structural information in an image, $S_\alpha$ [159] is used to assess the structural similarity between the regional perception ($S_\mathrm{r}$) and object perception ($S_\mathrm{o}$). Thus, $S_\alpha$ can be defined by

$$S_\alpha = \alpha S_\mathrm{o} + (1-\alpha)\, S_\mathrm{r} \tag{4}$$

where $\alpha \in [0, 1]$ is a weight. We set $\alpha = 0.5$ as the default, as suggested by Fan et al. [159].

**E-measure** ($E_\phi$). $E_\phi$ [160] was proposed based on cognitive vision studies to capture image-level statistics and local pixel matching information. Thus, $E_\phi$ can be defined by

$$E_\phi = \frac{1}{WH}\sum_{i=1}^{W}\sum_{i=1}^{H}\phi_{\mathrm{FM}}(i,j) \tag{5}$$

where $\phi_{\mathrm{FM}}$ denotes the enhanced-alignment matrix [160].

### 5.2 Performance comparison and analysis

#### 5.2.1 *Overall evaluation*

To quantify the performance of different models, we conducted a comprehensive evaluation of 24 representative RGB-D based salient object detection models, including nine traditional methods: LHM [51], ACSD [56], DESM [49], GP [50], LBE [57], DCMC [36], SE [37], CDCP [84], CDB [95], and fifteen deep learning-based methods: DF [52], PCF [92], CTMF [58], CPFP [53], TANet [103], AFNet [106], MMCI [55], DMRA [54], D$^3$Net [38], SSF [39], A2dele [40], S$^2$MA [41], ICNet [42], JL-DCF [43], and UC-Net [44]. We report the mean values of $S_\alpha$ and MAE across the five datasets (STERE [139], NLPR [51] , LFSD [140], DES [49], and SIP [38]) for each model in Fig. 5. Better models appear in the upper left corner (i.e., with larger $S_\alpha$ and smaller MAE). From Fig. 5, we may make following observations:

- *Traditional versus deep learning models.* Compared to traditional RGB-D based salient object detection models, deep learning methods obtain significantly better performance. This confirms the powerful feature learning ability of deep networks.

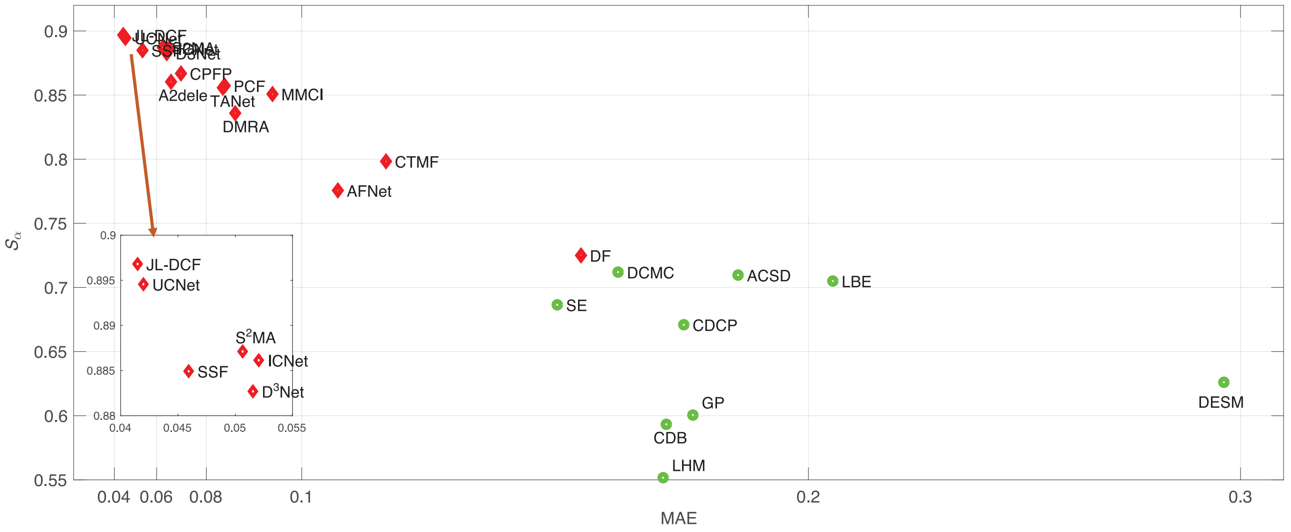

**Fig. 5** Comprehensive evaluation of 24 representative RGB-D based salient object detection models: LHM [51], ACSD [56], DESM [49], GP [50], LBE [57], DCMC [36], SE [37], CDCP [84], CDB [95], DF [52], PCF [92], CTMF [58], CPFP [53], TANet [103], AFNet [106], MMCI [55], DMRA [54], D$^3$Net [38], SSF [39], A2dele [40], S$^2$MA [41], ICNet [42], JL-DCF [43], and UC-Net [44]. For each, we report the mean values of $S_\alpha$ and MAE across five datasets: STERE [139], NLPR [51], LFSD [140], DES [49], and SIP [38]. Better models appear in the upper left corner (i.e., with larger $S_\alpha$ and smaller MAE). Red diamonds: deep models. Green circles: traditional models.

- *Comparison of deep models.* Among the deep learning-based models, $D^3$Net [38], JL-DCF [43], UC-Net [44], SSF [39], ICNet [42], and $S^2$MA [41] obtain the best performance.

Figures 6 and 7 show PR and F-measure curves for the 24 representative RGB-D based salient object detection models, for eight datasets: STERE [139], NLPR [51], LFSD [140], DES [49], SIP [38], GIT [47], SSD [85] , and NJUD [56]). Note that there are 1000, 300, 100, 135, 929, and 80 test samples for NLPR, LFSD, DES, SIP, GIT, and SSD, respectively. For the NJUD [56] dataset, there are 485 test images for CPFP [53], $S^2$MA [41], ICNet [42], JL-DCF [43], and UC-Net [44], and 498 testing images for all other models.

To understand the best six models in depth, we discuss their main advantages below.

$D^3$Net [38] consists of two key components, a three-stream feature learning module and a depth purifier unit. The three-stream feature learning module has three subnetworks: RgbNet, RgbdNet, and DepthNet. RgbNet and DepthNet are used to learn high-level feature representations for RGB and depth images, respectively, while RgbdNet is used to learn their fused representations. This three-stream feature learning module can capture modality-specific information as well as the correlation between modalities. Balancing the two aspects is very important for multi-modal learning and helps to improve the salient object detection performance. The depth purifier unit acts as a gate to explicitly remove low-quality depth maps, whose effects other existing methods often do not consider. Because low-quality depth maps can hinder fusion of RGB images and depth maps, the depth purifier unit can ensure effective multi-modal fusion to achieve robust salient object detection.

JL-DCF [43] has two key components, for joint learning (JL) and densely-cooperative fusion (DCF).

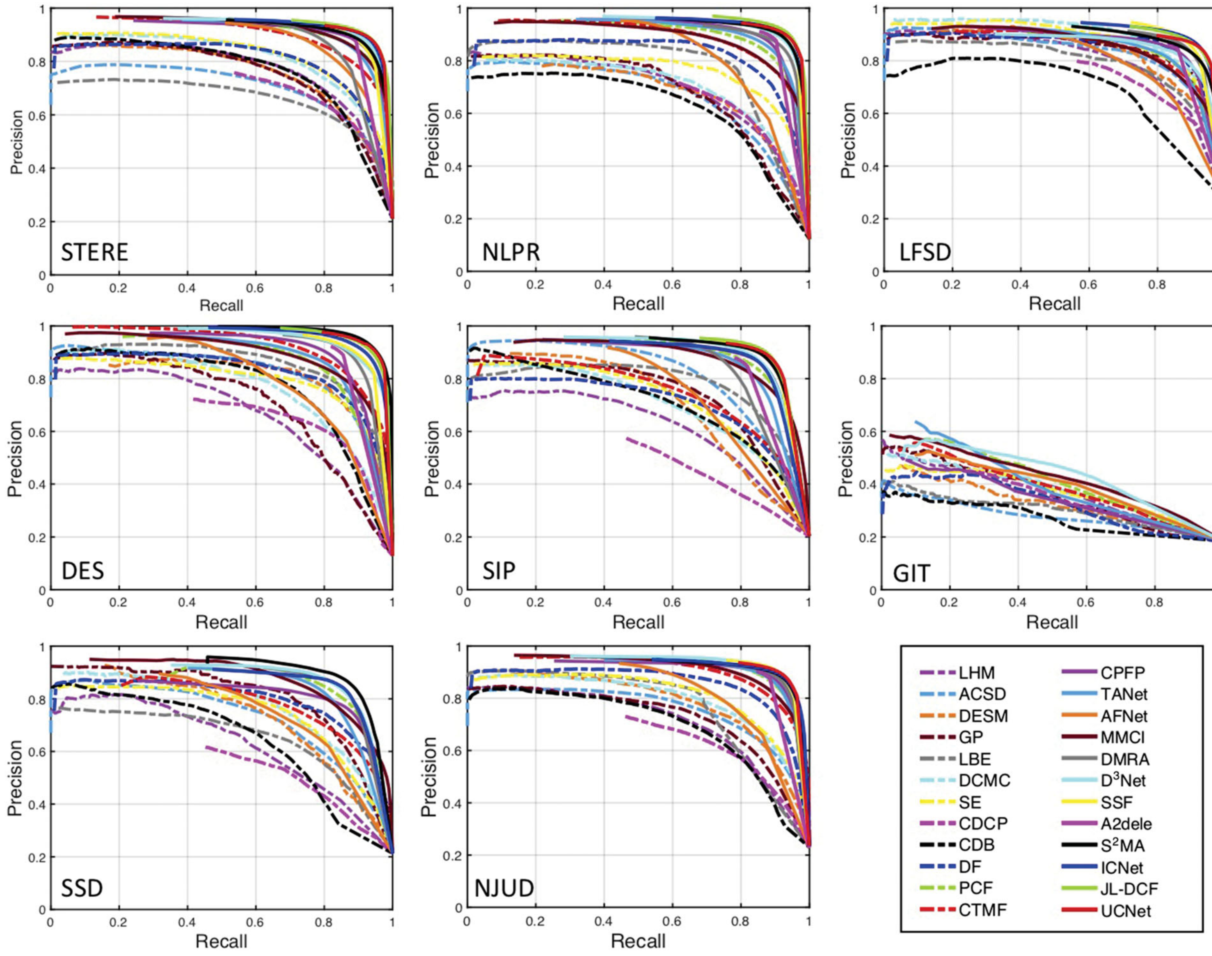

**Fig. 6** PR curves for 24 RGB-D based models, for the STERE [139], NLPR [51], LFSD [140], DES [49], SIP [38], GIT [47], SSD [85], and NJUD [56] datasets.

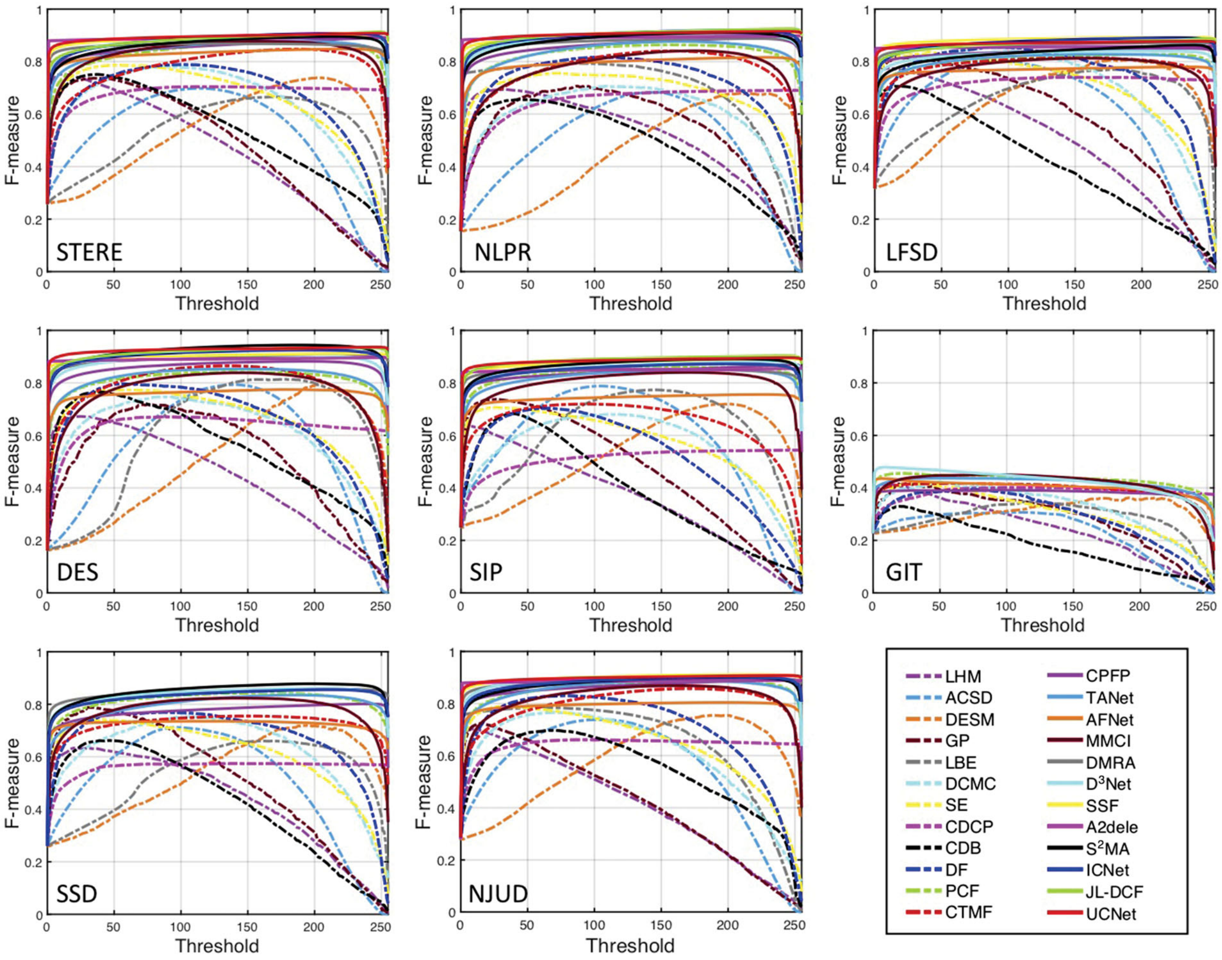

**Fig. 7** F-measures under different thresholds for 24 RGB-D based models on the STERE [139], NLPR [51], LFSD [140], DES [49], SIP [38], GIT [47], SSD [85], and NJUD [56] datasets.

Specifically, the JL module is used to learn robust saliency features, while the DCF module is used for complementary feature discovery. This method uses a middle-fusion strategy to extract deep hierarchical features from RGB images and depth maps, in which cross-modal complementarity is effectively exploited to achieve accurate prediction.

UC-Net [44], instead of producing a single saliency prediction, produces multiple predictions by modeling the distribution of the feature output space as a generative model conditioned on RGB-D images. Because each person has specific preferences in labeling a saliency map, the stochastic characteristic of saliency may not be captured when a single saliency map is produced for an image pair using a deterministic learning pipeline. The strategy in this model can take into account human uncertainty in saliency annotation. Moreover, depth maps can suffer from noise. Directly fusing RGB images and depth maps can cause the network to fit this noise. Therefore, a depth correction network, designed as an auxiliary component, is used to refine depth information with a semantic guided loss. All of these key components help to improve salient object detection performance.

In SSF [39], a complementary interaction module (CIM) is developed to explore discriminative cross-modal complementarity and to fuse cross-modal features, where region-wise attention is introduced to supplement rich boundary information for each modality. A compensation-aware loss is used to improve the network's confidence for hard samples in unreliable depth maps. These key components enable the proposed model to effectively explore and establish the complementarity of cross-modal feature representations, while at the same time reducing the negative effects of low-quality depth maps, boosting salient object detection performance.

ICNet [42] uses an information conversion module to interactively and adaptively explore correlations between high-level RGB and depth features. A cross-modal depth-weighted combination block is introduced to enhance the differences between the RGB and depth features at each level, ensuring that the features are treated differently. ICNet exploits the complementarity of cross-modal features, as well as exploring continuity of cross-level features, both of which help to achieve accurate predictions.

$S^2$MA [41] uses a self-mutual attention module (SAM) to fuse RGB and depth images, integrating self-attention and mutual attention to propagate context more accurately. The SAM can provide additional complementary information from multi-modal data to improve salient object detection performance, overcoming the limitations of only using self-attention, i.e., a single modality. To reduce the effects of low-quality depth cues (due to e.g., noise), a selection mechanism is used to reweight the mutual attention. This can filter out unreliable information, resulting in more accurate saliency prediction.

#### 5.2.2 *Attribute-based evaluation*

To investigate the influence of different factors, such as object scale, background clutter, number of salient objects, indoor or outdoor scene, background objects, and lighting conditions, we carried out diverse attribute-based evaluations on several representative RGB-D based salient object detection models.

**Object scale**. To characterize the scale of a salient object, we compute the ratio of the size of the salient area to that of the whole image. We define three object scales: small, when the ratio is less than 0.1, large, when the ratio is greater than 0.4, and medium, otherwise. For this evaluation, we built a hybrid dataset with 2464 images collected from STERE [139], NLPR [51] , LFSD [140], DES [49], and SIP [38], where 24%, 69.2%, and 6.8% of images have small, medium, and large salient objects respectively. The constructed hybrid dataset can be found at https://github.com/taozh2017/RGBD-SODsurvey. Some sample images with objects of different scales are shown in Fig. 8. The results of the attribute-based comparison w.r.t. object scale are shown in Table 8. It can be observed that all methods perform best at detecting small salient objects and worst for large salient objects. The three most recent models: JL-DCF [43], UC-Net [44], and $S^2$MA [41], achieve the best performance. $D^3$Net [38], SSF [39], A2dele [40], and ICNet [42] also obtain promising performance.

**Background clutter**. It is difficult to directly characterize background clutter. Since classic salient object detection methods tend to use prior information or color contrast to locate salient objects, they often fail in the presence of complex backgrounds.

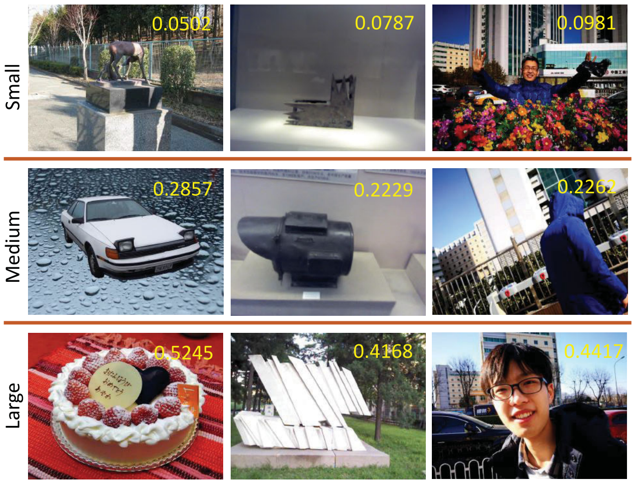

**Fig. 8** Images with objects at different scales. Scale ratios are given in yellow.

**Table 8** Attribute-based study w.r.t. salient object scales. 24 representative RGB-D based salient object detection models (9 traditional, 15 deep learning-based) are compared in terms of MAE and $S_\alpha$. The three best results are shown in red, blue, and green

| | | Traditional models | | | | | | | | | Deep learning-based models | | | | | | | | | | | | | | |
|---|---|---|---|---|---|---|---|---|---|---|---|---|---|---|---|---|---|---|---|---|---|---|---|---|---|
| | Scale | LHM [51] | ACSD [56] | DESM [49] | GP [50] | LBE [57] | DCMC [36] | SE [37] | CDCP [84] | CDB [95] | DF [52] | PCF [92] | CTMF [58] | CPFP [53] | TANet [103] | AFNet [106] | MMCI [55] | DMRA [54] | $D^3$Net [38] | SSF [39] | A2dele [40] | $S^2$MA [41] | ICNet [42] | JL-DCF [43] | UC-Net [44] |
| MAE | Small | 0.065 | 0.149 | 0.319 | 0.098 | 0.177 | 0.108 | 0.056 | 0.128 | 0.073 | 0.087 | 0.042 | 0.065 | 0.044 | 0.041 | 0.046 | 0.051 | 0.030 | 0.033 | 0.031 | 0.032 | 0.035 | 0.036 | 0.032 | 0.034 |
| | Medium | 0.178 | 0.183 | 0.287 | 0.180 | 0.210 | 0.158 | 0.150 | 0.173 | 0.179 | 0.152 | 0.068 | 0.107 | 0.055 | 0.067 | 0.095 | 0.079 | 0.069 | 0.053 | 0.045 | 0.054 | 0.052 | 0.052 | 0.041 | 0.042 |
| | Large | 0.403 | 0.311 | 0.310 | 0.377 | 0.261 | 0.305 | 0.364 | 0.308 | 0.385 | 0.310 | 0.112 | 0.183 | 0.093 | 0.118 | 0.213 | 0.130 | 0.181 | 0.102 | 0.105 | 0.114 | 0.088 | 0.104 | 0.085 | 0.072 |
| | Overall | 0.166 | 0.184 | 0.296 | 0.173 | 0.206 | 0.156 | 0.142 | 0.171 | 0.167 | 0.147 | 0.065 | 0.102 | 0.055 | 0.065 | 0.091 | 0.076 | 0.067 | 0.052 | 0.046 | 0.053 | 0.051 | 0.052 | 0.041 | 0.042 |
| $S_\alpha$ | Small | 0.624 | 0.668 | 0.517 | 0.650 | 0.645 | 0.700 | 0.775 | 0.661 | 0.666 | 0.745 | 0.847 | 0.789 | 0.840 | 0.846 | 0.792 | 0.832 | 0.860 | 0.879 | 0.876 | 0.859 | 0.877 | 0.882 | 0.881 | 0.883 |
| | Medium | 0.543 | 0.732 | 0.658 | 0.598 | 0.723 | 0.727 | 0.676 | 0.683 | 0.585 | 0.730 | 0.863 | 0.805 | 0.877 | 0.862 | 0.779 | 0.859 | 0.838 | 0.888 | 0.893 | 0.865 | 0.893 | 0.892 | 0.906 | 0.901 |
| | Large | 0.386 | 0.630 | 0.686 | 0.450 | 0.731 | 0.604 | 0.479 | 0.586 | 0.424 | 0.597 | 0.838 | 0.761 | 0.855 | 0.827 | 0.682 | 0.830 | 0.734 | 0.846 | 0.837 | 0.815 | 0.863 | 0.845 | 0.859 | 0.876 |
| | Overall | 0.552 | 0.710 | 0.626 | 0.601 | 0.705 | 0.712 | 0.686 | 0.671 | 0.593 | 0.725 | 0.857 | 0.798 | 0.867 | 0.856 | 0.776 | 0.851 | 0.836 | 0.883 | 0.885 | 0.860 | 0.887 | 0.886 | 0.897 | 0.895 |

Thus, in this evaluation, we utilize five traditional salient object detection methods: BSCA [161], CLC [162], MDC [163], MIL [164], and WFD [165], to first detect salient objects in various images, and then categorise these images as having simple or complex backgrounds according to the results. Specifically, we first constructed a hybrid dataset with 1400 images collected from three datasets (STERE [139], NLPR [51], and LFSD [140]). Then, we applied the five models to this dataset and obtained $S_\alpha$ values for each image, which we used to characterize images as follows. If all $S_\alpha$ values are higher than 0.9, the image is considered to have a simple background. If all $S_\alpha$ values are lower than 0.6, the image is said to have a complex background. The remaining images are deemed to be uncertain. Some example images with these three types of background clutter are shown in Fig. 9. The constructed hybrid dataset can be found at https://github.com/taozh2017/RGBD-SODsurvey. The results of the attribute-based comparison w.r.t. background clutter are shown in Table 9. All models are worse at salient object detection for images with complex backgrounds than simple ones. Among the representative models, JL-DCF [43], UC-Net [44], and SSF [39] achieve the three best results. The four most recent models: D$^3$Net [38], S$^2$MA [41], A2dele [40], and ICNet [42], obtain better performance than the other models.

**Single and multiple objects**. For this evaluation, we constructed a hybrid dataset with 1229 images from the NLPR [51] and SIP [38] datasets. Some example images with single and multiple salient objects are shown in Fig. 10. The comparison results are shown in Fig. 11. From the results, we can see that it is easier to detect single salient object than multiple ones.

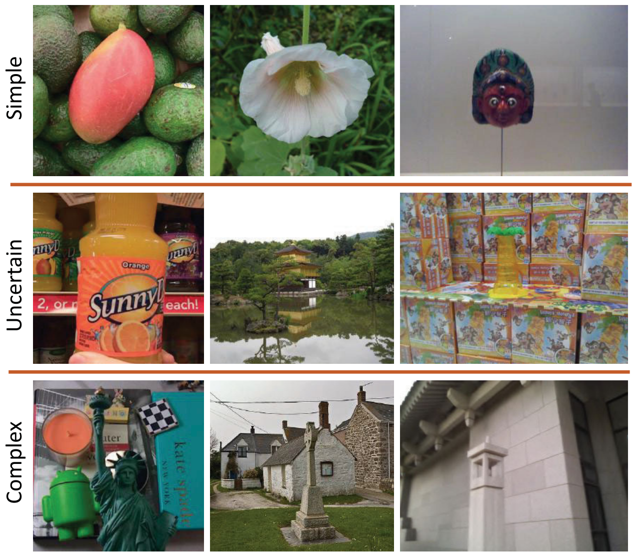

**Fig. 9** Images with three types of background clutter.

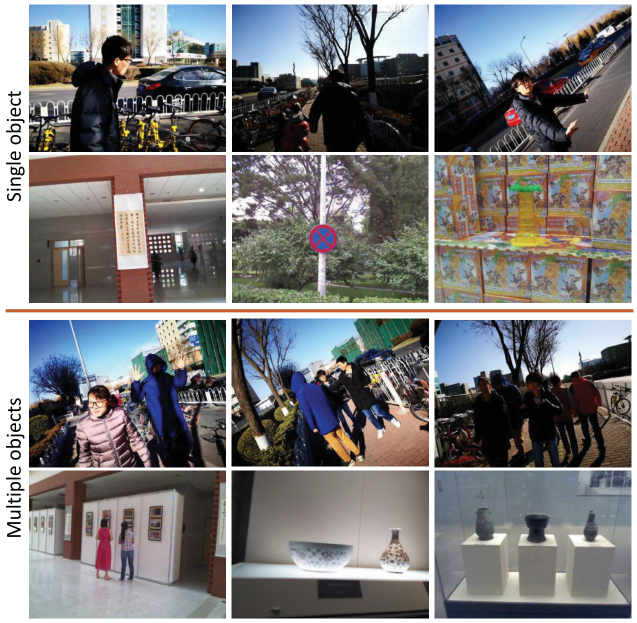

**Fig. 10** Images with single or multiple salient objects.

**Table 9** Attribute-based study w.r.t. background clutter. 24 representative RGB-D based salient object detection models (9 traditional, 15 deep learning-based) are compared in terms of MAE and $S_\alpha$. The three best results are shown in red, blue, and green

| | | Traditional models | | | | | | | | | Deep learning-based models | | | | | | | | | | | | | | |
|---|---|---|---|---|---|---|---|---|---|---|---|---|---|---|---|---|---|---|---|---|---|---|---|---|---|
| | background | LHM [51] | ACSD [56] | DESM [49] | GP [50] | LBE [57] | DCMC [36] | SE [37] | CDCP [84] | CDB [95] | DF [52] | PCF [92] | CTMF [58] | CPFP [53] | TANet [103] | AFNet [106] | MMCI [55] | DMRA [54] | D$^3$Net [38] | SSF [39] | A2dele [40] | S$^2$MA [41] | ICNet [42] | JL-DCF [43] | UC-Net [44] |
| MAE | Simple | 0.100 | 0.163 | 0.219 | 0.150 | 0.202 | 0.056 | 0.084 | 0.028 | 0.136 | 0.045 | 0.031 | 0.053 | 0.018 | 0.033 | 0.031 | 0.041 | 0.028 | 0.017 | 0.012 | 0.010 | 0.016 | 0.013 | 0.014 | 0.013 |
| | Uncertain | 0.164 | 0.195 | 0.294 | 0.175 | 0.210 | 0.140 | 0.133 | 0.139 | 0.159 | 0.129 | 0.062 | 0.081 | 0.050 | 0.059 | 0.075 | 0.070 | 0.058 | 0.045 | 0.043 | 0.043 | 0.049 | 0.041 | 0.037 | 0.037 |
| | Complex | 0.159 | 0.190 | 0.349 | 0.180 | 0.205 | 0.190 | 0.147 | 0.236 | 0.143 | 0.163 | 0.085 | 0.110 | 0.079 | 0.077 | 0.108 | 0.094 | 0.087 | 0.071 | 0.065 | 0.070 | 0.072 | 0.079 | 0.063 | 0.065 |
| | Overall | 0.160 | 0.193 | 0.295 | 0.174 | 0.209 | 0.140 | 0.132 | 0.141 | 0.157 | 0.127 | 0.063 | 0.082 | 0.051 | 0.059 | 0.076 | 0.070 | 0.059 | 0.046 | 0.043 | 0.043 | 0.049 | 0.043 | 0.038 | 0.038 |
| $S_\alpha$ | Simple | 0.781 | 0.787 | 0.761 | 0.694 | 0.748 | 0.930 | 0.856 | 0.941 | 0.704 | 0.944 | 0.944 | 0.913 | 0.958 | 0.937 | 0.922 | 0.933 | 0.935 | 0.960 | 0.966 | 0.965 | 0.965 | 0.969 | 0.961 | 0.962 |
| | Uncertain | 0.572 | 0.694 | 0.638 | 0.606 | 0.695 | 0.736 | 0.723 | 0.727 | 0.610 | 0.774 | 0.873 | 0.853 | 0.882 | 0.873 | 0.818 | 0.868 | 0.854 | 0.900 | 0.894 | 0.884 | 0.895 | 0.910 | 0.909 | 0.907 |
| | Complex | 0.496 | 0.627 | 0.509 | 0.545 | 0.616 | 0.577 | 0.605 | 0.487 | 0.575 | 0.627 | 0.782 | 0.742 | 0.787 | 0.790 | 0.694 | 0.768 | 0.751 | 0.822 | 0.815 | 0.786 | 0.813 | 0.808 | 0.829 | 0.833 |
| | Overall | 0.576 | 0.693 | 0.633 | 0.606 | 0.691 | 0.732 | 0.720 | 0.718 | 0.612 | 0.770 | 0.869 | 0.847 | 0.878 | 0.869 | 0.813 | 0.863 | 0.850 | 0.896 | 0.891 | 0.879 | 0.892 | 0.904 | 0.904 | 0.904 |

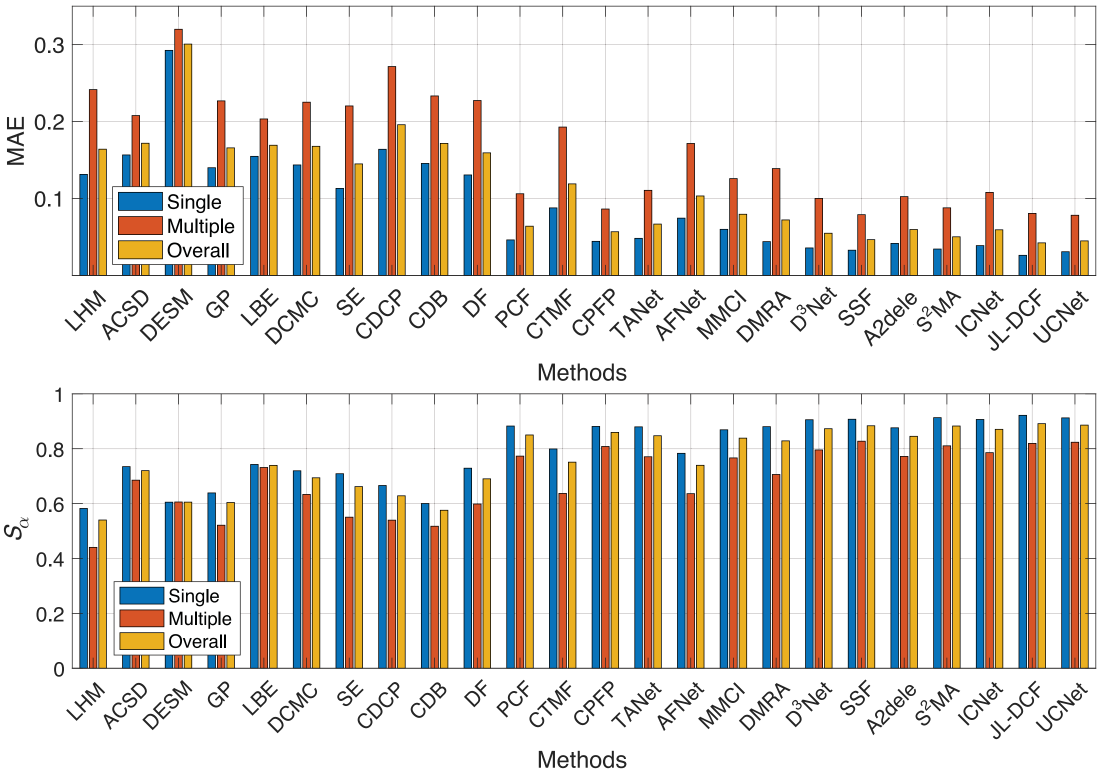

**Fig. 11** Attribute-based study w.r.t. number of salient objects (single or multiple). Comparative results for 24 representative RGB-D based salient object detection models: LHM [51], ACSD [56], DESM [49], GP [50], LBE [57], DCMC [36], SE [37], CDCP [84], CDB [95], DF [52], PCF [92], CTMF [58], CPFP [53], TANet [103], AFNet [106], MMCI [55], DMRA [54], D$^3$Net [38], SSF [39], A2dele [40], S$^2$MA [41], ICNet [42], JL-DCF [43], and UC-Net [44] in terms of MAE (above) and $S_\alpha$ (below).

**Indoors and outdoors**. We evaluated the performance of different RGB-D based salient object detection models on indoor and outdoor scenes. For this evaluation, we constructed a hybrid dataset collected from the DES [49], NLPR [51], and LFSD [140] datasets. The results are shown in Fig. 12. It can be seen that most models struggle more to detect salient objects in indoor scene than outdoor scenes. This is possibly because indoor environments often have varying lighting conditions.

**Background objects**. We evaluated the performance of RGB-D based salient object detection models in the presence of different backgrounds. We used the SIP dataset [38], and split it into eight categories: car, barrier, flower, grass, road, sign, tree, and other. The results of the comparison are shown in Table 10. All methods obtain diverse performances with different background objects. Among the 24 representative RGB-D based models, JL-DCF [43], UC-Net [44], and SSF [39] achieve the three best results. The four most recent models, i.e., D$^3$Net [38], S$^2$MA [41], A2dele [40], and ICNet [42] obtain better performance than the others.

**Lighting conditions**. The performance of salient object detection methods can be affected by the lighting conditions. To determine the effects on different RGB-D based salient object detection models, we conducted an evaluation on the SIP dataset [38], whose images we split into two categories: sunny and low-light. The results of the comparison are shown in Table 11. Low light negatively impacts salient object detection performance. Among the models compared, UC-Net [44] obtained the best performance under sunny conditions, while JL-DCF [43] achieved the best result under low light.

**Visual comparison**. We further report saliency maps generated for various challenging scenes to allow visualization of the performance of different RGB-D based salient object detection models. Figures 13 and 14 show some representative examples for two classic non-deep methods: DCMC [36] and SE [37], and eight state-of-the-art CNN-based models: DMRA [54], D$^3$Net [38], SSF [39], A2dele [40], S$^2$MA [41], ICNet [42], JL-DCF [43], and UC-Net [44]. Row 1 shows a small object, while row 2 shows a large object. Rows 3 and 4 contain complex backgrounds

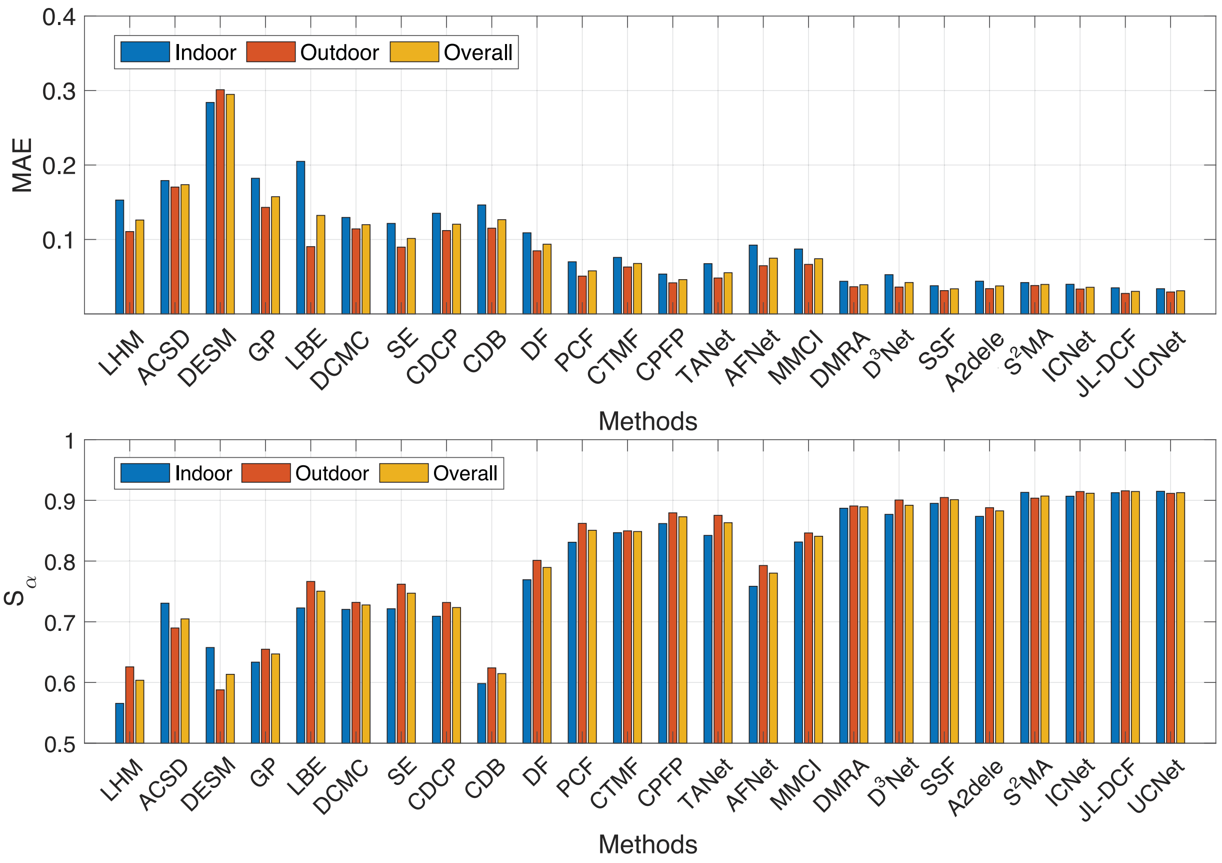

**Fig. 12** Attribute-based study w.r.t. indoor vs. outdoor environments. Comparative results for 24 representative RGB-D based salient object detection models: LHM [51], ACSD [56], DESM [49], GP [50], LBE [57], DCMC [36], SE [37], CDCP [84], CDB [95], DF [52], PCF [92], CTMF [58], CPFP [53], TANet [103], AFNet [106], MMCI [55], DMRA [54], D$^3$Net [38], SSF [39], A2dele [40], S$^2$MA [41], ICNet [42], JL-DCF [43], and UC-Net [44] in terms of MAE (above) and $S_\alpha$ (below).

**Table 10** Attribute-based study w.r.t. background objects: car, barrier, flower, grass, road, sign, tree, and other. The methods compared including 24 representative RGB-D based salient object detection models (9 traditional and 15 deep learning-based) evaluated on the SIP dataset [38] in terms of MAE and $S_\alpha$. The three best results are shown in red, blue, and green

| | | Traditional models | | | | | | | | | Deep learning-based models | | | | | | | | | | | | | | |
|---|---|---|---|---|---|---|---|---|---|---|---|---|---|---|---|---|---|---|---|---|---|---|---|---|---|
| | Categories | LHM [51] | ACSD [56] | DESM [49] | GP [50] | LBE [57] | DCMC [36] | SE [37] | CDCP [84] | CDB [95] | DF [52] | PCF [92] | CTMF [58] | CPFP [53] | TANet [103] | AFNet [106] | MMCI [55] | DMRA [54] | D$^3$Net [38] | SSF [39] | A2dele [40] | S$^2$MA [41] | ICNet [42] | JL-DCF [43] | UC-Net [44] |
| MAE | Car | 0.158 | 0.163 | 0.301 | 0.159 | 0.201 | 0.185 | 0.154 | 0.202 | 0.171 | 0.171 | 0.085 | 0.134 | 0.094 | 0.084 | 0.101 | 0.093 | 0.069 | 0.061 | 0.063 | 0.078 | 0.055 | 0.067 | 0.058 | 0.057 |
| | Barrier | 0.197 | 0.177 | 0.308 | 0.180 | 0.201 | 0.196 | 0.176 | 0.251 | 0.203 | 0.202 | 0.073 | 0.149 | 0.060 | 0.078 | 0.128 | 0.089 | 0.093 | 0.068 | 0.054 | 0.074 | 0.057 | 0.075 | 0.052 | 0.053 |
| | Flower | 0.105 | 0.122 | 0.306 | 0.099 | 0.186 | 0.158 | 0.063 | 0.141 | 0.101 | 0.132 | 0.091 | 0.075 | 0.133 | 0.100 | 0.090 | 0.081 | 0.046 | 0.095 | 0.107 | 0.051 | 0.104 | 0.025 | 0.054 | 0.075 |
| | Grass | 0.164 | 0.161 | 0.279 | 0.155 | 0.184 | 0.167 | 0.138 | 0.182 | 0.176 | 0.167 | 0.041 | 0.110 | 0.035 | 0.048 | 0.088 | 0.059 | 0.056 | 0.037 | 0.030 | 0.046 | 0.033 | 0.043 | 0.023 | 0.029 |
| | Road | 0.189 | 0.167 | 0.281 | 0.176 | 0.187 | 0.181 | 0.164 | 0.225 | 0.189 | 0.169 | 0.070 | 0.140 | 0.054 | 0.072 | 0.125 | 0.078 | 0.093 | 0.059 | 0.049 | 0.072 | 0.050 | 0.065 | 0.045 | 0.044 |
| | Sign | 0.107 | 0.126 | 0.268 | 0.110 | 0.184 | 0.126 | 0.079 | 0.134 | 0.118 | 0.096 | 0.058 | 0.101 | 0.063 | 0.060 | 0.077 | 0.083 | 0.051 | 0.055 | 0.051 | 0.054 | 0.048 | 0.054 | 0.050 | 0.057 |
| | Tree | 0.192 | 0.193 | 0.310 | 0.190 | 0.241 | 0.194 | 0.183 | 0.230 | 0.219 | 0.205 | 0.083 | 0.157 | 0.083 | 0.091 | 0.132 | 0.109 | 0.106 | 0.083 | 0.067 | 0.074 | 0.092 | 0.097 | 0.063 | 0.071 |
| | Other | 0.246 | 0.217 | 0.329 | 0.224 | 0.229 | 0.216 | 0.229 | 0.274 | 0.233 | 0.233 | 0.106 | 0.177 | 0.111 | 0.111 | 0.170 | 0.124 | 0.140 | 0.095 | 0.083 | 0.099 | 0.100 | 0.100 | 0.084 | 0.086 |
| | Overall | 0.184 | 0.172 | 0.298 | 0.173 | 0.200 | 0.186 | 0.164 | 0.224 | 0.192 | 0.185 | 0.071 | 0.139 | 0.064 | 0.075 | 0.118 | 0.086 | 0.085 | 0.063 | 0.053 | 0.070 | 0.057 | 0.069 | 0.049 | 0.051 |
| $S_\alpha$ | Car | 0.516 | 0.731 | 0.590 | 0.603 | 0.714 | 0.671 | 0.591 | 0.613 | 0.546 | 0.631 | 0.811 | 0.726 | 0.786 | 0.807 | 0.736 | 0.813 | 0.817 | 0.856 | 0.845 | 0.804 | 0.870 | 0.846 | 0.855 | 0.859 |
| | Barrier | 0.497 | 0.727 | 0.609 | 0.575 | 0.728 | 0.672 | 0.612 | 0.553 | 0.552 | 0.643 | 0.837 | 0.698 | 0.860 | 0.831 | 0.708 | 0.830 | 0.792 | 0.855 | 0.874 | 0.821 | 0.871 | 0.848 | 0.876 | 0.875 |
| | Flower | 0.477 | 0.775 | 0.573 | 0.673 | 0.703 | 0.707 | 0.772 | 0.667 | 0.639 | 0.750 | 0.771 | 0.738 | 0.714 | 0.760 | 0.688 | 0.785 | 0.824 | 0.789 | 0.768 | 0.845 | 0.804 | 0.901 | 0.856 | 0.811 |
| | Grass | 0.537 | 0.756 | 0.643 | 0.605 | 0.760 | 0.728 | 0.683 | 0.672 | 0.559 | 0.672 | 0.908 | 0.770 | 0.908 | 0.899 | 0.780 | 0.888 | 0.876 | 0.917 | 0.924 | 0.878 | 0.928 | 0.910 | 0.939 | 0.924 |
| | Road | 0.521 | 0.739 | 0.634 | 0.598 | 0.751 | 0.685 | 0.641 | 0.595 | 0.576 | 0.680 | 0.851 | 0.722 | 0.871 | 0.848 | 0.705 | 0.847 | 0.807 | 0.873 | 0.885 | 0.832 | 0.885 | 0.868 | 0.889 | 0.892 |
| | Sign | 0.578 | 0.786 | 0.634 | 0.628 | 0.719 | 0.745 | 0.761 | 0.714 | 0.615 | 0.757 | 0.855 | 0.756 | 0.833 | 0.857 | 0.771 | 0.818 | 0.848 | 0.849 | 0.849 | 0.842 | 0.871 | 0.861 | 0.859 | 0.840 |
| | Tree | 0.505 | 0.699 | 0.606 | 0.577 | 0.661 | 0.648 | 0.600 | 0.588 | 0.543 | 0.625 | 0.802 | 0.679 | 0.804 | 0.778 | 0.691 | 0.779 | 0.748 | 0.806 | 0.837 | 0.807 | 0.800 | 0.788 | 0.848 | 0.825 |
| | Other | 0.460 | 0.687 | 0.594 | 0.532 | 0.706 | 0.669 | 0.563 | 0.554 | 0.542 | 0.600 | 0.786 | 0.677 | 0.774 | 0.782 | 0.647 | 0.790 | 0.722 | 0.800 | 0.828 | 0.785 | 0.809 | 0.799 | 0.821 | 0.823 |
| | Overall | 0.511 | 0.732 | 0.616 | 0.588 | 0.727 | 0.683 | 0.628 | 0.595 | 0.557 | 0.653 | 0.842 | 0.716 | 0.850 | 0.835 | 0.720 | 0.833 | 0.806 | 0.860 | 0.874 | 0.828 | 0.872 | 0.854 | 0.880 | 0.875 |

**Table 11** Attribute-based study w.r.t. light conditions (sunny vs. low-light). The comparison methods include 24 representative RGB-D based salient object detection models (9 traditional models and 15 deep learning-based models) evaluated on the SIP dataset [38] in terms of MAE and $S_\alpha$. The three best results are shown in red, blue, and green fonts

| | | Traditional models | | | | | | | | | Deep learning-based models | | | | | | | | | | | | | | |
|---|---|---|---|---|---|---|---|---|---|---|---|---|---|---|---|---|---|---|---|---|---|---|---|---|---|
| | Conditions | LHM [51] | ACSD [56] | DESM [49] | GP [50] | LBE [57] | DCMC [36] | SE [37] | CDCP [84] | CDB [95] | DF [52] | PCF [92] | CTMF [58] | CPFP [53] | TANet [103] | AFNet [106] | MMCI [55] | DMRA [54] | $D^3$Net [38] | SSF [39] | A2dele [40] | $S^2$MA [41] | ICNet [42] | JL-DCF [43] | UC-Net [44] |
| MAE | Sunny | 0.182 | 0.171 | 0.294 | 0.171 | 0.200 | 0.183 | 0.160 | 0.218 | 0.190 | 0.181 | 0.069 | 0.137 | 0.062 | 0.075 | 0.116 | 0.085 | 0.083 | 0.062 | 0.052 | 0.068 | 0.057 | 0.068 | 0.048 | 0.051 |
| | Low-light | 0.198 | 0.178 | 0.323 | 0.187 | 0.201 | 0.207 | 0.193 | 0.268 | 0.208 | 0.211 | 0.078 | 0.154 | 0.073 | 0.076 | 0.130 | 0.091 | 0.103 | 0.067 | 0.059 | 0.080 | 0.058 | 0.081 | 0.059 | 0.055 |
| | Overall | 0.184 | 0.172 | 0.298 | 0.173 | 0.200 | 0.186 | 0.164 | 0.224 | 0.192 | 0.185 | 0.071 | 0.139 | 0.064 | 0.075 | 0.118 | 0.086 | 0.085 | 0.063 | 0.053 | 0.070 | 0.057 | 0.069 | 0.049 | 0.051 |
| $S_\alpha$ | Sunny | 0.516 | 0.733 | 0.622 | 0.593 | 0.728 | 0.690 | 0.639 | 0.607 | 0.560 | 0.660 | 0.843 | 0.718 | 0.852 | 0.834 | 0.723 | 0.833 | 0.811 | 0.861 | 0.875 | 0.831 | 0.872 | 0.856 | 0.882 | 0.876 |
| | low-light | 0.481 | 0.721 | 0.573 | 0.554 | 0.722 | 0.635 | 0.556 | 0.515 | 0.543 | 0.610 | 0.838 | 0.701 | 0.838 | 0.837 | 0.700 | 0.832 | 0.775 | 0.855 | 0.867 | 0.810 | 0.871 | 0.839 | 0.867 | 0.871 |
| | Overall | 0.511 | 0.732 | 0.616 | 0.588 | 0.727 | 0.683 | 0.628 | 0.595 | 0.557 | 0.653 | 0.842 | 0.716 | 0.850 | 0.835 | 0.720 | 0.833 | 0.806 | 0.860 | 0.874 | 0.828 | 0.872 | 0.854 | 0.880 | 0.875 |

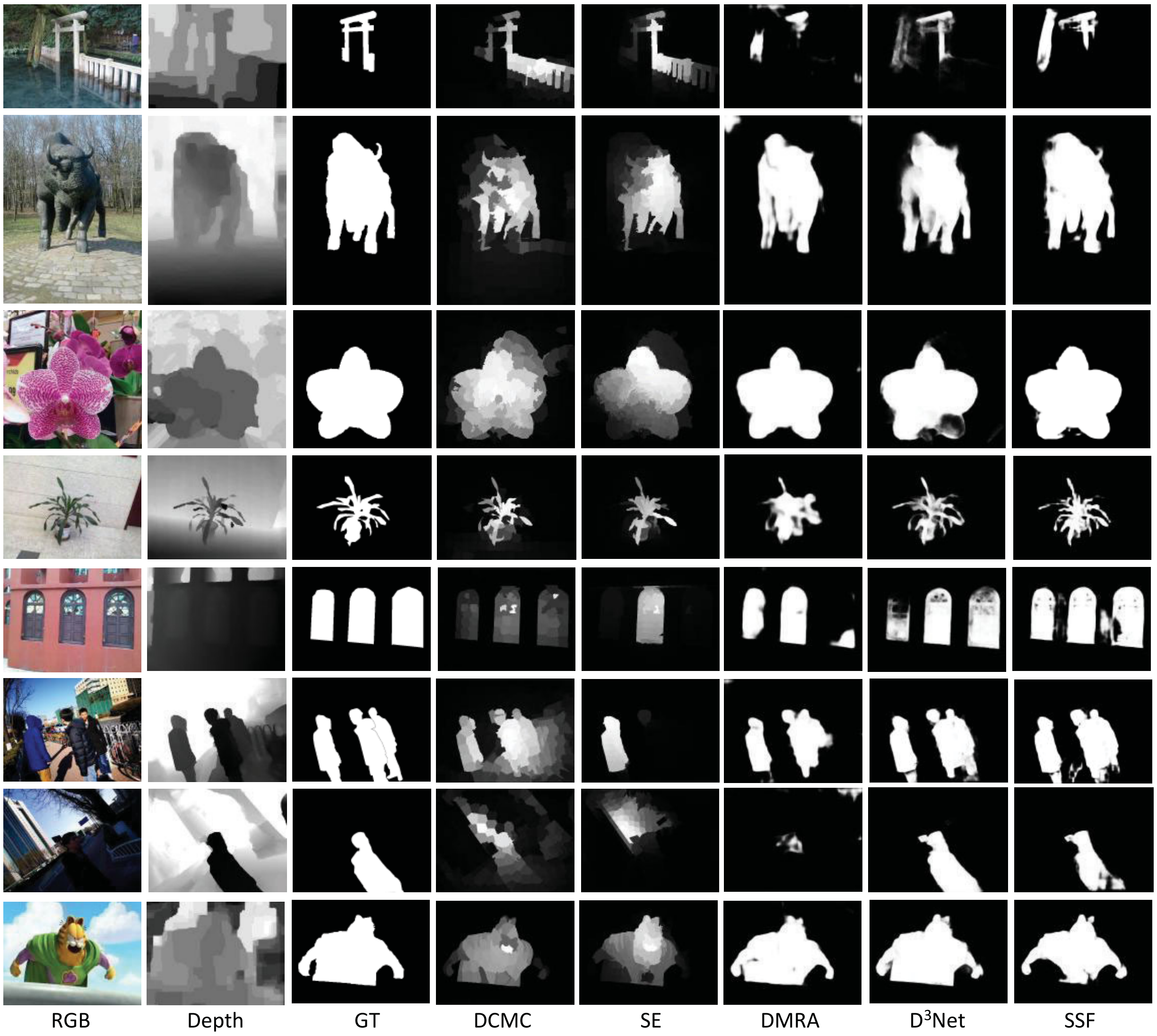

**Fig. 13** Visual comparison of two classical non-deep methods: DCMC [36] and SE [37], and three state-of-the-art CNN-based models: DMRA [54], $D^3$Net [38], SSF [39].

and boundaries respectively. Rows 5 and 6 contain multiple salient objects. Row 7 has low light. Row 8 has a coarse depth map with very inaccurate object boundaries, which could degrade salient object detection performance. It can be observed that deep models perform better than non-deep models on these challenging scenes, confirming the power of deep features over handcrafted ones. $D^3$Net [38], $S^2$MA

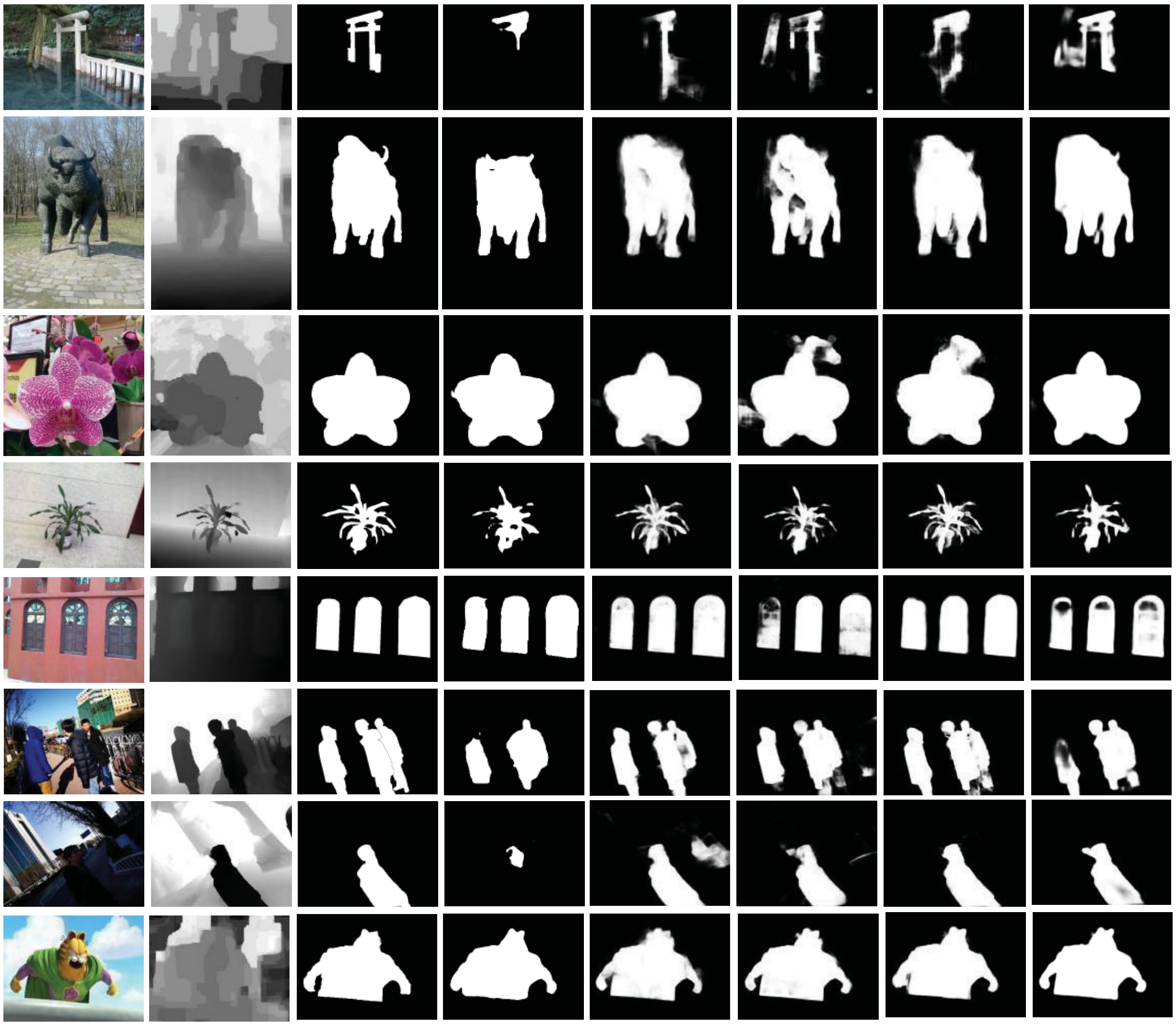

**Fig. 14** Visual comparison of five state-of-the-art CNN-based models: A2dele [40], $S^2$MA [41], ICNet [42], JL-DCF [43], and UC-Net [44].

[41], JL-DCF [43], and UC-Net [44] perform better than other deep models.

## 6 Challenges and open directions

### 6.1 Effects of imperfect depth

#### *6.1.1 Effects of low-quality depth maps*

Depth maps with detailed spatial information have proven beneficial in detecting salient objects against cluttered backgrounds, while the depth quality directly affects salient object detection performance. The quality of depth maps varies tremendously across different scenarios due to the nature of depth sensors, posing a challenge when trying to reduce the effects of low-quality depth maps. However, most existing methods directly fuse RGB images and original raw data from depth maps, without considering the effects of low-quality depth maps. There are a few notable exceptions. For example, in Ref. [53], a contrast-enhanced network was proposed to learn enhanced depth maps, with much higher contrast than the original depths. In Ref. [39], a compensation-aware loss was designed to pay more attention to hard samples containing unreliable depth information. $D^3$Net [38] uses a depth purifier unit to classify depth maps as reasonable or low-quality. It also acts as a gate to filter out low-quality depth maps. However, such methods often employ a two-step strategy to achieve depth enhancement and multi-modal fusion [39, 53] or an independent gate operation to remove poor depths, which could lead to a suboptimal problem. There is thus a need to develop an end-to-end framework that can achieve depth enhancement or adaptively assign low weights to poor depth maps during multi-modal fusion, which would be more helpful in reducing the effects of low-quality depth maps and boosting salient object detection performance.

##### 6.1.2 Incomplete depth maps

In RGB-D datasets, it is inevitable for there to be some low-quality depth maps due to the limitations of the acquisition devices. As previously discussed, several depth enhancement algorithms have been used to improve the quality of depth maps. However, depth maps that suffer from severe noise or blurred edges are often discarded. In this case, we have complete RGB images but some samples without depth maps, which is similar to the incomplete multi-view modal learning problem [166–170]. We may call this problem *incomplete RGB-D based salient object detection*. As current models only focus on salient object detection using complete RGB images and depth maps, we believe this could be a new direction for RGB-D salient object detection.

##### 6.1.3 Depth estimation

Depth estimation provides an effective solution to recover high-quality depths and overcome the effects of low-quality depth maps. Various depth estimation approaches [171–174] have been developed, which could be introduced into the RGB-D based salient object detection task to improve performance.

#### 6.2 Effective fusion strategies

##### 6.2.1 Adversarial learning-based fusion

It is important to effectively fuse RGB images and depth maps for RGB-D based salient object detection. Existing models often employ different fusion strategies (early fusion, middle fusion, or late fusion) to exploit the correlations between RGB images and depth maps. Recently, generative adversarial networks (GANs) [175] have gained widespread attention for the saliency detection task [176, 177]. In common GAN-based salient object detection models, a generator takes RGB images as inputs and generates the corresponding saliency maps, while a discriminator determines whether a given image is synthetic or ground-truth. GAN-based models could easily be extended to RGB-D salient object detection, which could help to boosting performance due to their superior feature learning ability. Moreover, GANs could also be used to learn common feature representations for RGB images and depth maps [114], which could help with feature or saliency map fusion and further boost salient object detection performance.

##### 6.2.2 Attention-induced fusion

Attention mechanisms have been widely applied to various deep learning-based tasks [178–181], allowing networks to selectively pay attention to a subset of regions for extracting powerful and discriminative features. Co-attention mechanisms have also been developed to explore the underlying correlations between multiple modalities. They are widely studied in visual question answering [182, 183] and video object segmentation [184]. Thus, for the RGB-D based salient object detection task, we could also develop attention-based fusion algorithms to exploit correlations between RGB images and depth cues to improve the performance.

#### 6.3 Different supervision strategies

Existing RGB-D models often use a fully supervised strategy to learn saliency prediction models. However, annotating pixel-level saliency maps is a tedious and time-consuming procedure. To alleviate this issue, there has been increasing interest in weakly and semi-supervised learning, which have been applied to salient object detection [185–189]. Semi- and weak supervision could also be introduced into RGB-D salient object detection, by leveraging image-level tags [185] and pseudo pixel-wise annotations [188, 190], to improve detection performance. Furthermore, several studies [191, 192] have suggested that models pre-trained using self-supervision can effectively be used to achieve better performance. Therefore, we could train saliency prediction models on large amounts of annotated RGB images in a self-supervised manner and then transfer the pre-trained models to the RGB-D salient object detection task.

#### 6.4 Dataset collection

##### 6.4.1 Dataset size

Although there are nine public RGB-D datasets for salient object detection, their size is quite limited, with the largest, NJUD [56], containing about 2000 samples. When compared to other RGB-D datasets for generic object detection or action recognition [193, 194], the RGB-D datasets for salient object detection are very small. Thus, it is essential to develop new large-scale RGB-D datasets to serve as baselines for future research.

##### *6.4.2 Complex backgrounds & task-driven datasets*

Most existing RGB-D datasets contain images with one salient object, or multiple objects but against a relatively clean background. However, real-world applications often involve much more complicated situations, e.g., occlusion, appearance change, and low illumination, which can reduce salient object detection performance. Thus, collecting images with complex backgrounds is critical to improving the generalizability of RGB-D salient object detection models. Moreover, for some tasks, images with specific salient object(s) must be collected. For example, road sign recognition is important in driver assistance systems, requiring images with road signs to be collected. Thus, it is essential to construct task-driven RGB-D datasets like SIP [38].

#### 6.5 Model design for real-world scenarios

Some smart phones can capture depth maps (e.g., images in the SIP dataset were captured using a Huawei Mate10). Thus it is feasible to perform salient object detection for real-world applications on smart devices. However, most existing methods include complicated and deep DNNs to increase model capacity and for better performance, preventing them from being directly applied to such platforms. To overcome this, model compression [195, 196] techniques could be used to learn compact RGB-D based salient object detection models with promising detection accuracy. Moreover, JL-DCF [43] utilizes a shared network to locate salient objects using RGB and depth views, which largely reduces the model parameters and makes real-world applications feasible.

#### 6.6 Extension to RGB-T

In addition to RGB-D salient object detection, there are several other methods that fuse different modalities for better detection, such as RGB-T salient object detection, which integrates RGB and thermal infrared data. Thermal infrared cameras can capture the heat radiation emitted from any object, making thermal infrared images insensitive to illumination conditions [197]. Therefore, thermal images can provide supplementary information to improve salient object detection when images of salient objects suffer from varying light, glare, or shadows. Some RGB-T models [197–205] and datasets (VT821 [199], VT1000 [203], and VT5000 [205]) have already been proposed over the past few years. Like for RGB-D salient object detection, the key aim of RGB-T salient object detection is to fuse RGB and thermal infrared images and exploit the correlations between the two modalities. Thus, several advanced multi-modal fusion technologies in RGB-D salient object detection could be extended to the RGB-T salient object detection task.

### 7 Conclusions

This paper has presented the first comprehensive review of RGB-D based salient object detection models. We have reviewed the models from different perspectives, and summarized popular RGB-D salient object detection datasets as well as providing details of each. As light fields also provide depth information, we have also reviewed popular light field salient object detection models and related benchmark datasets. We have comprehensively evaluated 24 representative RGB-D based salient object detection models, as well as performing an attribute-based evaluation based on new datasets. Moreover, we have discussed several challenges and highlighted open directions for future research. In addition, we have briefly discussed the extension to RGB-T salient object detection to improve robustness to lighting conditions. Although RGB-D based salient object detection has made notable progress over the past several decades, there is still significant room for improvement. We hope this survey will generate more interest in this field.

### Acknowledgements

This research was supported by a Major Project for a New Generation of AI under Grant No. 2018AAA0100400, National Natural Science Foundation of China (61922046), and Tianjin Natural Science Foundation (17JCJQJC43700).
